# Refining the Ambush Hypothesis: Evidence That GC- and AT-Rich Bacteria Employ Different Frameshift Defence Strategies

**DOI:** 10.1093/gbe/evy075

**Published:** 2018-04-02

**Authors:** Liam Abrahams, Laurence D Hurst

**Affiliations:** Department of Biology and Biochemistry, The Milner Centre for Evolution, University of Bath, United Kingdom

**Keywords:** out-of-frame stop codon, dual coding, sequence evolution, ambush hypothesis, frameshift

## Abstract

Stop codons are frequently selected for beyond their regular termination function for error control. The “ambush hypothesis” proposes out-of-frame stop codons (OSCs) terminating frameshifted translations are selected for. Although early indirect evidence was partially supportive, recent evidence suggests OSC frequencies are not exceptional when considering underlying nucleotide content. However, prior null tests fail to control amino acid/codon usages or possible local mutational biases. We therefore return to the issue using bacterial genomes, considering several tests defining and testing against a null. We employ simulation approaches preserving amino acid order but shuffling synonymous codons or preserving codons while shuffling amino acid order. Additionally, we compare codon usage in amino acid pairs, where one codon can but the next, otherwise identical codon, cannot encode an OSC. OSC frequencies exceed expectations typically in AT-rich genomes, the +1 frame and for TGA/TAA but not TAG. With this complex evidence, simply rejecting or accepting the ambush hypothesis is not warranted. We propose a refined post hoc model, whereby AT-rich genomes have more accidental frameshifts, handled by RF2–RF3 complexes (associated with TGA/TAA) and are mostly +1 (or −2) slips. Supporting this, excesses positively correlate with in silico predicted frameshift probabilities. Thus, we propose a more viable framework, whereby genomes broadly adopt one of the two strategies to combat frameshifts: preventing frameshifting (GC-rich) or permitting frameshifts but minimizing impacts when most are caught early (AT-rich). Our refined framework holds promise yet some features, such as the bias of out-of-frame sense codons, remain unexplained.

## Introduction

DNA sequences have the ability to carry multiple overlapping layers of noncoding, yet critical “dual-coding” information. Examples are widespread (Itzkovitz et al. 2010; Lin et al. 2011; Shabalina et al. 2013; Pancsa and Tompa 2016) often preventing or mitigating the cellular costs of transcriptional or translational errors (Drummond and Wilke 2009; Warnecke and Hurst 2011). The highly diverse nature of errors means signatures of dual-coding error control mechanisms are also varied. For instance, codon and amino acid usage is biased toward exon ends as purifying selection acts at synonymous and nonsynonymous sites of exonic splice enhancers (ESEs; Parmley et al. 2006,^2007^; Wu and Hurst 2015) to minimize mis-splicing rates (Blencowe 2000; Fairbrother et al. 2004; Wu et al. 2005; Caceres and Hurst 2013). Similarly, codon usage biases are thought to minimize translational missense errors (Drummond and Wilke 2008; Zhou et al. 2009; Serohijos et al. 2012), while synonymous and nonsynonymous site evolution in nucleosome linker sequences governs correct nucleosome positioning (Warnecke et al. 2008). Furthermore, synonymous codon selection surrounding micro-RNA (miRNA) binding sites ensures efficient miRNA binding (Gu et al. 2012).

Alternatively, avoiding particular sequences or motifs may be of equal importance. Selection acts to prevent mutations that cause inappropriate binding of RNA-binding proteins’ binding within coding sequences (CDSs; Savisaar and Hurst 2017), to avoid intra-CDS Shine-Dalgarno (SD) motifs (Shine and Dalgarno 1974) that limit synthesis rates and promote incorrect folding inducing undesired frameshifting (Betney et al. 2010; Li et al. 2012; Diwan and Agashe 2016), or to avoid mononucleotide repeats or sequences prone to ribosomal slippage (Ackermann and Chao 2006; Gurvich et al. 2005; Gu et al. 2010a).

Beyond their principle termination function, stop codons are repeatedly implicated in error control. In-frame stop codons located in introns are under selection (He et al. 1993; Jaillon et al. 2008; Farlow et al. 2010; Mekouar et al. 2010) to allow nonsense-mediated decay (NMD) to selectively degrade incorrectly spliced transcripts. In CDS regions where NMD is unable to operate, codons in close nucleotide space proximity to a stop codon are selectively avoided as a robustness to mistranscription errors (Cusack, et al. 2011). Stop codons found 5’ to recognized translation initiation sites increase protein activity, suggesting unwanted or incorrect translation initiations prior to the recognized start codon are terminated. (Seligmann 2007).

Despite selection to mitigate translational errors, the trade-off between optimal decoding accuracy and translational speed (Wohlgemuth et al. 2010) permits ribosomal frameshifts errors, synthesizing peptides never intended. Robustness to such errors is thought to drive selection on transport RNA (tRNA) repertoires in genomes where frameshifts may be more costly (Warnecke et al. 2010) and may direct ribosome evolution (Atkins and Bjork 2009). Further, the ability to correct frameshift errors is thought to explain why three stop codons exist (Itzkovitz and Alon 2007). Out-of-frame stop codons (OSCs) prematurely terminate frameshifted translation events, minimizing process and cytotoxic costs associated with synthesizing an incorrect peptide from the incorrect reading frame (cellular resources, unproductive ribosomal demand, and toxic aggregation; Gingold and Pilpel 2011).

Recently, we identified a strong site-specific signature of selection for one OSC (Abrahams and Hurst 2017), finding a significant excess of A at CDSs fourth sites in nearly all bacterial genomes. Translation initiation on an ATG (and more generally, NTG) that becomes +1 out of frame thus encounters TGA, providing the potential ability for immediate ribosome correction. The “ambush hypothesis” (Seligmann and Pollock 2004), however, proposes that OSCs should be selectively favored throughout the gene body to reduce genome-wide frameshift costs. Several studies examine usage of codons that could, but don’t necessarily, constitute an OSC and claim codon usage biases are consistent with such OSC selection (Seligmann and Pollock 2004; Singh and Pardasani 2009). However, with few genomes demonstrating biases (38.00%/6.23% of total genomes, 36.96%/7.07% of bacterial genomes for the two studies respectively), evidence is underwhelming. Moreover, these codon usage biases might be explained almost entirely by GC content (Morgens et al. 2013)—GC3 and GC1 content are the strongest determinants of OSC frequency in the +1 and +2 frames, respectively (Wong et al. 2008). Importantly, this method does not examine actual OSC frequencies. Thus, initial evidence supporting the ambush hypothesis is weak, speculative, and not robust to compositional controls to account for the high AT-content of stop codons.

An alternative approach compares real sequences with a distribution of null sequences simulating real CDSs, for which compositional biases can be controlled. Using Markov chain models, a remarkable 99.1% and 93.3% of prokaryotic genomes exhibit OSC excesses using second-order and fifth-order models that control for GC content and dinucleotide or pentanucleotide frequencies (Tse, et al. 2010), although numbers are reduced slightly for Morgens, et al. (2013) (83% and 85% respectively). Critically, these models directly interrogate OSC densities, although they do not preserve amino acid or codon usages.

While results from these models are consistent with OSCs exerting a near-universal selection pressure constraining CDS evolution, it is important to consider the wider biological context of these excesses. If the ambush hypothesis correctly predicts selection, prima facie it has been argued that selection to incorporate OSCs should be stronger in GC-rich genomes, as codon usage biases restrict chance dicodons yielding OSCs (note stop codons are AT-rich) (Tse et al. 2010; Morgens et al. 2013). Significant positive correlations between genome GC content and extent of excess suggests this is the case (Tse et al. 2010; Morgens et al. 2013). Yet, these excesses are attributable predominantly to TGA and not TAA or TAG (Morgens et al. 2013). Furthermore, out-of-frame sense TGN codons have similar, if not greater, number of genomes with excess and positive correlations with GC content (Morgens et al. 2013). These issues raise several potential caveats that may also apply to previous studies. First, when considered together, any excess may, for reasons unknown, only reflect TGA excesses, highlighting the need to consider each stop codon separately. Second, any excesses of OSCs might be an artifact of selection for codons with similar nucleotide composition and not selection directly for OSCs themselves, with OSC frequencies not exceeding expectations given underlying nucleotide composition.

The current status of the ambush hypothesis could therefore be considered as confused and uncertain with contradictory (i.e., some supportive and some unsupportive) evidence. Although the Markov models by Tse et al. (2010) and Morgens et al. (2013) improve on initial methods, are the results limited by the model design? As reported earlier, it is essential that GC content is controlled. Equally, as protein coding sequences are being simulated, the requirement for specific amino acids in specific orders might need to be retained. While the Markov models do provide some compositional bias control (GC content, higher order biases, e.g., dinucleotide frequencies), the stepwise addition of nucleotides does not preserve codon or amino acid identities, amino acid sequence ordering likely essential for protein function, nor small mutational or motif biases. Thus, the flexibility allowed by Markov models may not appropriately reflect real biological coding constraints that underpin OSC frequencies.

In this study, we therefore return to this issue concerning OSC selection. We first confirm previous results using Markov models (in part to ascertain whether our data set can mimic prior results). We then propose and test a series of simulation models that attempt to control for these compositional biases to varying degrees. While it is easy to criticize the Markov models, we acknowledge that our models also do not control completely for all competing selection pressures and biases.

In addition to the above mentioned problems, there is also the issue in quantifying deviation from null. We suppose a Z-score metric (deviation in standard deviation units) enables a more biologically valuable metric, as this enables us to quantify and compare excesses between models while accounting for genome variability. As +1 and −2 and +2 and −1 frameshifts incur equal costs (except for immediately at the start codon), for simulation models we consider only +1 and +2 frameshifts.

We find a complex pattern of results that provides neither a clear rejection nor acceptance of the ambush hypothesis. In this context, we motivate a post hoc refined version of the hypothesis, which broadly proposes that GC- and AT-rich genomes handle the problems associated with frameshifts differently, that +1 frameshifts are the dominant form of accidental slippage, and that frameshifts are predominantly resolved via a release factor (RF) 2/RF3 mechanism (which does not apply to TAG). In silico evidence supports the first tenet of the refined model, but we highlight several features that still defy clear explanation.

## Materials and Methods

### General Methods

All analyses were performed using custom Python 3.6 scripts with standard NumPy 1.8.0, SciPy 0.13, and Biopython 1.66 (Cock et al. 2009) libraries. Statistical analyses and data visualizations were performed using R 3.3.3 (R Core Team 2015). Scripts can be found at (https://github.com/la466/oscs).

### Genome Downloads and Filtering

Whole-genome sequences for 3,860 bacterial genomes were downloaded from the European Molecular Biology Laboratory (EBML) database (http://www.ebi.ac.uk/Tools/dbfetch/emblfetch?db=embl, last accessed January 19, 2017). Genomes were filtered to include only one genome per genus larger than 500,000 base pairs (the remaining genomes were not considered in the analysis) in order to minimize any biases attributable to phylogenetic nonindependence, leaving 694 genomes. Of these genomes, 690 use National Centre for Biotechnology Information (NCBI) translation tables 11 and 4 use NCBI translation table 4.

### Coding Sequence Filtering

Each coding sequence was subjected to filtering in order to ensure the integrity of the sequences analyzed. Sequences were limited to those that contained a multiple of three nucleotides, contained only A, C, G, or T nucleotides, contained no in-frame stop codons, and had a correctly defined stop codon according to the NCBI translation table, TAA, TAG, or TGA for table 11 genomes or TAA or TAG for table 4 genomes.

### General Modeling

All simulations were repeated 200 times for each bacterial genome. Increasing the number of simulations had minimal impact on OSC density variance (see supplementary fig. 1, Supplementary Material online, for an example of the variation in *Escherichia coli* OSC densities in the codon shuffle model). We define codon excesses using the standard Z score to compare how the real OSC densities differ beyond those expected by simulation between genomes while accounting for genome coding properties. *P* values were calculated by extrapolating directly from genome Z scores and corrected for multiple comparisons using the Benjamini-Hochberg False Discovery Rate (FDR) correction method, with one *P* value reported per genome. Where we report N/694 genomes with significant excesses, these are N different genomes with both genome Z > 0 and *P* < 0.05. OSC densities were calculated per 100 codons.

### Markov Models

For each genome, we built Markov models similar to Tse et al. (2010) and Morgens et al. (2013). For each CDS in the genome, start and stop codons were discounted. For second-order models, the first two nucleotides of the remaining sequence and their position in the codon were defined. The third nucleotide, given the previous two nucleotides and their codon positions, was then sampled. After each sample, the two seed nucleotides and codon positions were shifted one nucleotide and resampled until all nucleotides in all CDSs had been accounted for. For fifth-order models, samples were based on the previous five nucleotides. Each real CDS was simulated using the start codon and two or five seed nucleotides using the transition probabilities previously calculated until the simulated sequence was of the same length as the real CDS minus the stop codon, which was then appended.

### Codon Shuffle Model

For each CDS within the genome, the start and stop codons were removed. The codons of the CDS were isolated and randomly shuffled before being concatenated to form the simulated sequence.

### Synonymous Site Model

For each genome, nucleotide frequencies at synonymous sites of codons within each coding block were calculated and normalized within coding blocks. In contrast to the synonymous codon model, only synonyms within the same coding block were allowed to vary, and thus it is only the synonymous site that this model is questioning (e.g., serine AGC and AGT and TCA, TCC, TCG, and TCT are considered separately). Each codon in the real CDS had genome, amino acid, and coding block specific probabilities during simulation. For each CDS, each codon was in turn simulated using these coding probabilities.

### Synonymous Codon Model

For each genome, codon frequencies were calculated and normalized as the probability of encoding an amino acid. Codons from multiple coding blocks that encode the same amino acid were considered together. For each CDS, each codon was in turn simulated using these probabilities. This test therefore asks whether CDSs using preferentially uses synonymous codons that generate OSCs.

### Comparison between Table 11 and Table 4 Genomes

A local regression model (loess) for the specific codon and reading frame was fit between GC content and OSC density per 100 codons that included all table 11 and table 4 genomes in order to account for variation in GC content between the genomes. Residuals from this model for table 11 and table 4 genomes were then compared using Kruskal–Wallis tests. To increase the sample size, genomes of 89 additional table 4 genomes discarded during the original phylogenetic filter (irrespective of genome size) were considered for further comparison of OSC densities (see supplementary table 1, Supplementary Material online, for breakdown). These genomes were subjected to CDS filtering as before. We also restricted this table 4 genome data set by ranking *Mycoplasma* genomes by Z scores of +1 TGA for simulations using the synonymous site simulation and including only the nine genomes with highest Z score (matching the number of *Spiroplasma*, the next most common genus). Thus, this restriction should include only *Mycoplasma* genomes with the weakest negative TGA selection.

### Calculating Frameshift Costs and Probabilities

Information regarding tRNA isoacceptor copy number and diversity was downloaded from the tRNADB-CE (Abe 2011; last accessed October 30, 2017). Of our 694 genomes, tRNA copy number and diversity information was available for 281 genomes. As in Warnecke et al. (2010), only genomes in which each codon could be decoded by the tRNA repertoire were considered, resulting in a final set of 231 genomes.

The “genomic cost of processing model” (Warnecke et al. 2010, equation 1) was used to calculated the cost of accidental frameshifting. This model is nested to allow the calculation of the probability of individual codons frameshifting using equation 2 (Warnecke et al. 2010). We inherit the assumption that tRNA copy numbers are reasonable proxies for cellular tRNA concentrations (Dong et al. 1996; Kanaya et al. 1999; Cognat et al. 2008). Further, anticodon–codon matching strategies were derived using the Supplementary Methods from Warnecke et al. (2010) originally proposed by Grosjean et al. (2010).

### Codon Adaptation Index Calculations

Bacterial codon use is nonrandom. Highly expressed genes often prefer to use codons that are decoded by the most abundant tRNA (Rocha 2004). The Codon Adaptation Index (CAI) (Sharp and Li 1987) quantifies codon bias with high CAI values correlating with high expression in several organisms including *E coli* (dos Reis et al. 2003). CAI is therefore used as a gene expression proxy.

For each genome, a reference set of 20 genes from *rplA/1—rplF/6*, *rplI/9—rplU/21* and *rpsB/2*—*rpsU/21* were identified as highly expressed. The first 30 nucleotides were removed from the CDS (the 5’ CDS is biased to facilitate ribosome binding), and the first half of the CDS in this highly expressed set was used to calculate CAI indices using CodonW v1.4.4 (https://sourceforge.net/projects/codonw/; last accessed March 22, 2016) with the arguments “-coa_cu -coa_num 100%” to include all sequences in calculating indices. CAI values for the first half (minus the first 30 nucleotides) of the remaining CDS in the genome were calculated with the “-all_indices” argument using the generated fop_file, cai_file, and cbi_file. OSC densities were subsequently calculated using the second half of the CDS to prevent resampling of the same sequence for two measures for which codon usage is being measured and maximizing the independence of the data.

## Results

### Markov Models Replicate Prior OSC Excesses

To establish that our set of genomes is comparable with prior efforts, we first simulated sequences using Markov models in order to replicate prior results. Results demonstrate similar distributions of excesses to Morgens et al. (2013) (supplementary result 1, Supplementary Material online). The conclusions of prior results are repeatable, not consistent with ambush hypothesis predictions and that our sample of genomes are able to mimic prior efforts. Further discrepancies are therefore unlikely to be owing to the employment of a different set of genomes.

### Genomes with Significant OSC Excesses Are Predominantly AT-Rich in a Model in Which Real Codon Combinations Are Shuffled

It is potentially important that the amino acid content of the protein coding sequences is maintained during simulations. Assuming selection on nonsynonymous sites is stronger than on synonymous sites (Hurst 2009), the principle determinant of any codon is likely the amino acid it encodes. However, not all sense codons can yield an OSC; in order to generate an OSC, two conducive codons must combine in the correct order. A proportion of OSCs will be incorporated irrespective of OSC selection, given some chance dicodon pairs always yield an OSC. For example, any A-starting codon following a methionine codon generates A +1 TGA. Can the OSC frequency be explained by random (no selection for OSCs) dicodon pairings? To test this hypothesis, we randomized codon order within each CDS to disrupt codon combinations that generate OSCs. This simulation controls for GC content exactly while preserving exact amino and codon identities and interactions between codon second and third sites. Amino acid order is not constrained.

We find that 124/694 (17.88%) of genomes have a significant excess of OSCs after randomization (*P* < 0.05, false discovery rate [FDR] correction), much reduced when compared with the Markov models both here and in the previous studies (Tse et al. 2010; Morgens et al. 2013). When each reading frame is considered independently, 367/694 (52.88%, *P* < 0.05, FDR correction) genomes have significant excesses in the +1 frame but many fewer, 101/694 (14.55%, *P* < 0.05, FDR correction) genomes, have significant excess in the +2 frame.

**Fig. 1.**
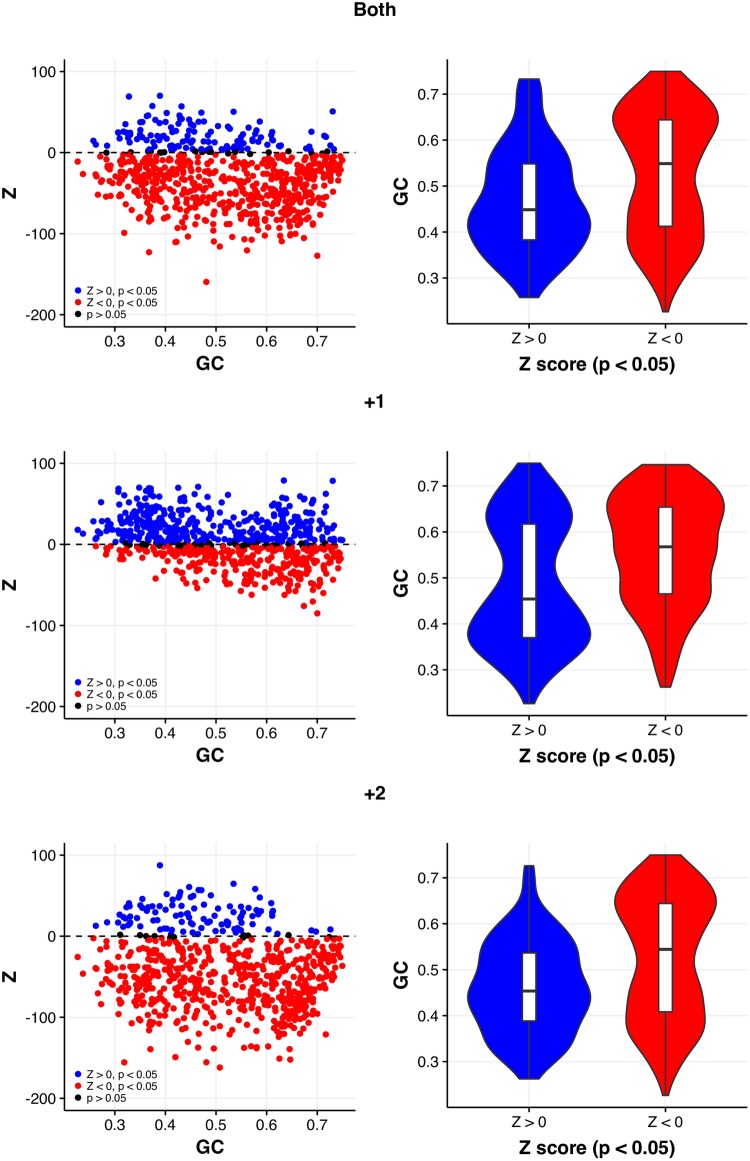
—Correlations between GC content and out-of-frame stop codon excess (Z > 0), when all stop codons are considered together, are significantly negative in each reading frame for coding sequences simulated by random codon shuffling within the CDS. Violin plots emphasize that excesses are biased toward AT-rich genomes.

While this evidence is suggestive of OSC selection in the +1 frame in some genomes, several unexpected features are notable. First, correlations between GC content and OSC excesses are significantly negative (Table 1). As post-frameshift runs are longer in GC-rich genomes, the opposite correlation might have been a more obvious prediction (and previously employed as a prediction by Tse et al. 2010 and Morgens et al. 2013). Second, we observe many genomes with significant negative excesses of OSCs (fig. 1), suggesting selection for OSCs is not ubiquitous and often avoided. Furthermore, positive excesses are predominantly limited to the +1 reading frame (fig. 1). Whether this reflects a possible preponderance and susceptibility to +1 frameshift events is unknown.

**Table 1:** The Number of Genomes with Significant Out-of-Frame Excesses in Alternative Reading Frames When Coding Sequences Have Been Simulated by Shuffling the Codons within the Coding Sequence. Spearman’s rank correlations between genome GC content and OSC excess, defined by the standard Z score, are also shown.

Codon	Reading Frame	# With Excess	% With Excess	ρ	*P*
All stops	Both	124	17.88	−0.178	2.328 × 10^−6^
All stops	+1	367	52.88	−0.295	2.664 × 10^−15^
All stops	+2	101	14.55	−0.144	1.489 × 10^−4^
TAA	Both	98	14.12	−0.427	<2.2 × 10^−16^
TAC	Both	168	24.21	−0.113	0.003
TAG	Both	118	17.00	−0.352	<2.2 × 10^−16^
TAT	Both	186	26.80	−0.343	<2.2 × 10^−16^
TGA	Both	353	50.86	−0.091	0.017
TGC	Both	599	86.31	0.498	<2.2 × 10^−16^
TGG	Both	281	40.49	−0.431	<2.2 × 10^−16^
TGT	Both	165	23.78	−0.308	1.436 × 10^−16^
TAA	+1	296	42.65	−0.417	<2.2 × 10^−16^
TAC	+1	361	52.02	0.572	<2.2 × 10^−16^
TAG	+1	190	27.38	−0.385	<2.2 × 10^−16^
TAT	+1	391	56.34	0.408	<2.2 × 10^−16^
TGA	+1	370	53.31	0.036	0.348
TGC	+1	575	82.85	0.465	<2.2 × 10^−16^
TGG	+1	256	36.89	−0.406	<2.2 × 10^−16^
TGT	+1	52	7.49	−0.063	0.099
TAA	+2	80	11.53	−0.231	8.587 × 10^−10^
TAC	+2	148	21.33	−0.404	<2.2 × 10^−16^
TAG	+2	44	6.34	−0.178	2.336 × 10^−6^
TAT	+2	176	25.36	−0.471	<2.2 × 10^−16^
TGA	+2	344	49.57	−0.169	7.508 × 10^−6^
TGC	+2	531	76.51	0.233	5.600 × 10^−10^
TGG	+2	299	43.08	−0.206	4.950 × 10^−8^
TGT	+2	362	52.16	−0.352	<2.2 × 10^−16^

Excesses of OSCs are also not uniformly distributed between the three stop codons. Only TGA has excesses in over 50% of genomes for any reading frame. This is also perhaps unexpected as TGA is thought to be the weakest of the stop codons (Povolotskaya et al. 2012; Korkmaz et al. 2014; Wei et al. 2016). TAA and TAG are often preferred and TGA avoided in highly expressed genes (Wei et al. 2016) while replacing TGA abolishes termination readthrough (Meng et al. 1995), implicating TGA as the least efficient terminator. A TGA preference was also observed by Morgens et al. (2013).

**Fig. 2.**
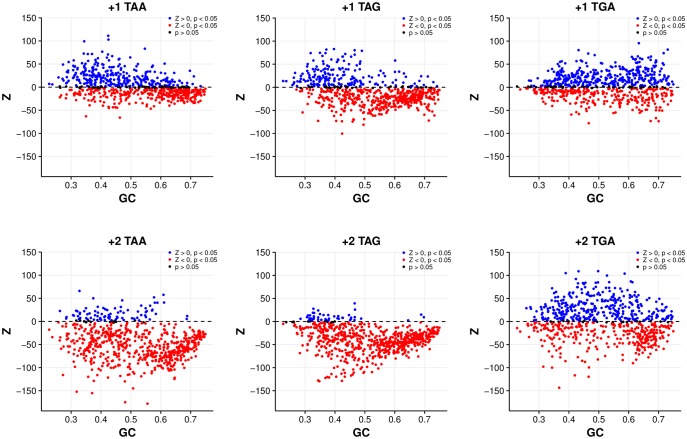
—Correlations between GC content and genome excess of out-of-frame stop codons (Z > 0) are significantly negative (*P* < 0.01, Spearman’s rank correlation) for all stop codons, in both reading frames, except for +1 TGA (*P *= 0.348) for the codon shuffle model. Excesses of TAA and TAG are heavily biased toward AT-rich genomes.

Genomes with significant excesses tend to be AT-rich, although significant TGA excesses do extend to some extremely GC-rich genomes, particularly in the +1 frame (fig. 2, Supplementary fig. 2, Supplementary Material online). Intriguingly, excesses of TAA and TAG are more highly restricted to AT-rich genomes, despite the identical GC content of TAG and TGA.

The observations of an excess of OSCs in some genomes in itself need not be evidence for selection for OSCs. Under the ambush hypothesis, we might also expect stronger selection for OSCs when compared with sense codons of similar nucleotide composition (Morgens et al. 2013). However, both TAC and TAT have a greater number of genomes with excesses when compared with TAA or TAG in both reading frames and excesses have significant positive correlations with GC content (Table 1). Excesses of +1 TGC have the strongest correlation and occur in the greatest percentage of genomes when compared with other TGN codons. By contrast, the number of genomes with excesses is greater for TGA than for either TGG or TGT in the +1 frame although only TGG in the +2 frame. Thus, as suggested by Morgens et al. (2013), OSC excesses may simply reflect complex compositional requirements resulting in an overrepresentation of out-of-frame TAN or TGN codons as opposed to selection for OSCs themselves.

### OSC Excesses Are Also Seen in a Null Model Where Synonymous Sites Are Randomized

The above mentioned model provided some evidence for an excess of OSCs, especially in AT-rich genomes, although this evidence is by no means unambiguous. There are, however, limitations with the form of the null model used above. Disruptive changes to amino acid sequences would fundamentally alter protein function and not be permitted during sequence evolution. Such disruption would also break up larger motifs. Similar to the Markov models, this model cannot account for site-specific amino acid selection. Indeed, changes to sensitive amino acids can induce conformational changes in protein structure, altering protein stability or robustness to mutational errors (Yutani et al. 1977, Hormoz 2013) and are therefore essential to protein function. Moreover, amino acids that may carry site-specific functional information, for example, the second amino acid that is under strong selection to promote methionine cleavage (Liao et al. 2004; Frottin et al. 2006; Ouidir, et al. 2015), are not retained.

A possibly more realistic scenario might be strong selection for synonymous mutations that generate OSCs. To consider this, we simulated synonymous nucleotide frequencies in accordance with genome codon usage frequencies preserving amino acid identities, amino acid order, and net genome codon usage frequencies. For these simulations, we permitted synonymous codon changes from strictly within the same codon block, i.e., codons from the 2-fold and 4-fold blocks of the three 6-fold degenerate amino acids were not interchanged. A similar but less stringent codon simulation model where this codon block restriction is relaxed (i.e., allowing the interchange of all members within 6-fold degenerate blocks) yields similar results (supplementary result 2, Supplementary Material online).

With higher level constraints controlled, if OSCs enforce a strong enough selection pressure, we expect a bias toward nucleotides generating OSCs if the following codon permits. For example, if the amino acid sequence dictates isoleucine-glutamic acid, we expect a bias toward ATA isoleucine codons to encode a +1 TAG. OSCs arising from 1-fold degenerates are not considered as synonymous site selection has no effect.

**Fig. 3.**
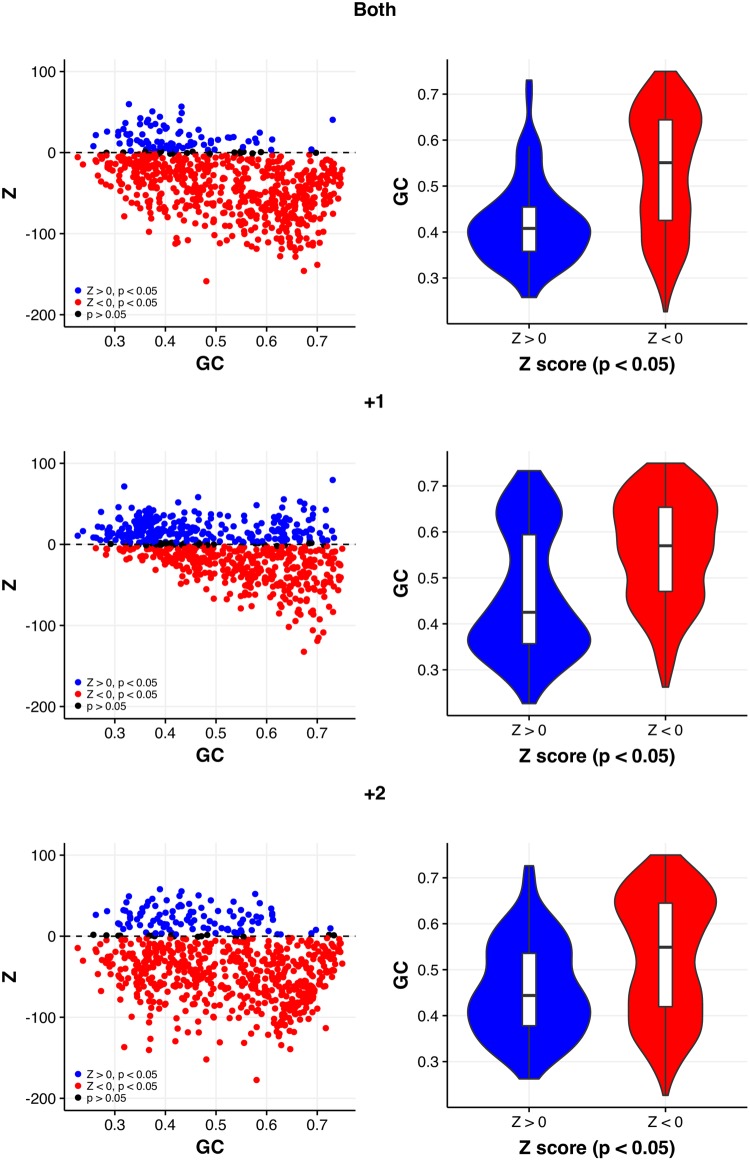
—Correlations between GC content and out-of-frame stop codon excess (Z > 0), when all stop codons are considered together, are significantly negative (*P* < 0.01, Spearman’s rank correlation) in each alternative reading frame for coding sequences where synonymous sites are randomized. Violin plots again emphasize a bias towards significant excesses in the AT-rich genomes.

Perhaps significantly, much like the previous model, the number of genomes with significant excesses is low and predominantly in the +1 frame (272/694, 39.19%, *P *< 0.05, FDR correction) (table 2). The lack of excesses in the +2 frame is particularly surprising for this model, given T is strictly required at the synonymous site for OSCs. When all OSCs are considered together, excesses in each reading frame are significantly negatively correlated with GC content (table 2) and heavily biased toward AT-rich genomes (fig. 3).

**Table 2:** The Number of Genomes with Significant Out-of-Frame Excesses for Different Codons When Coding Sequences Have Been Simulated by Randomizing Synonymous Sites within Coding Blocks. Spearman's rank correlations between genome GC content and OSC excess, defined by the standard Z score, are also shown

Codon	Reading Frame	# With Excess	% With Excess	ρ	*P*
All stops	Both	87	12.54	−0.444	<2.2 × 10^−16^
All stops	+1	272	39.19	−0.443	<2.2 × 10^−16^
All stops	+2	103	14.84	−0.260	4.046 × 10^−12^
TAA	Both	118	17.00	−0.508	<2.2 × 10^−16^
TAC	Both	145	20.89	−0.067	0.077
TAG	Both	101	14.55	−0.282	4.371 × 10^−14^
TAT	Both	194	27.95	−0.382	<2.2 × 10^−16^
TGA	Both	288	41.50	−0.326	< 2.2 × 10^−16^
TGC	Both	636	91.64	0.589	<2.2 × 10^−16^
TGG	Both	265	38.18	−0.404	<2.2 × 10^−16^
TGT	Both	252	36.31	−0.403	<2.2 × 10^−16^
TAA	+1	298	42.94	−0.444	<2.2 × 10^−16^
TAC	+1	330	47.55	0.595	<2.2 × 10^−16^
TAG	+1	155	22.33	−0.334	<2.2 × 10^−16^
TAT	+1	439	63.26	0.403	<2.2 × 10^−16^
TGA	+1	256	36.89	−0.135	3.729 × 10^−4^
TGC	+1	599	86.31	0.625	<2.2 × 10^−16^
TGG	+1	271	39.05	−0.365	<2.2 × 10^−16^
TGT	+1	98	14.12	−0.218	7.287 × 10^−9^
TAA	+2	93	13.40	−0.321	<2.2 × 10^−16^
TAC	+2	146	21.04	−0.389	<2.2 × 10^−16^
TAG	+2	42	6.05	−0.140	2.270 × 10^−4^
TAT	+2	185	26.66	−0.500	<2.2 × 10^−16^
TGA	+2	365	52.59	−0.261	3.777 × 10^−12^
TGC	+2	557	80.26	0.178	2.523 × 10^−6^
TGG	+2	271	39.05	−0.214	1.386 × 10^−8^
TGT	+2	384	55.33	−0.409	<2.2 × 10^−16^

This lack of significant excess extends to the individual OSCs. When both frames are considered together, TGA again demonstrates the greatest deviations from null sequences (288/694, 41.50%, *P *< 0.05, FDR correction). Excesses of TAA are lower (118/694, 17.00%, *P* < 0.05, FDR correction) and TAG lower still (101/694, 14.55%, *P *< 0.05, FDR correction). All OSC excesses are limited predominantly to AT-rich genomes (supplementary fig. 3, Supplementary Material online).

**Fig. 4.**
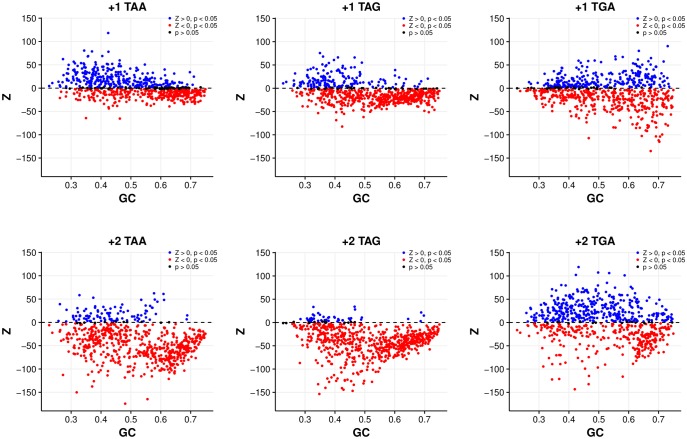
—Correlations between GC content and genome excess of out-of-frame stop codons (Z > 0) are significantly negative (*P* < 0.01, Spearman’s rank correlation) for all stop codons in both alternative reading frames for the synonymous site randomisation model. Excesses of TAA and TAG are heavily biased toward AT-rich genomes, with few genomes exhibiting excesses in the +2 frame.

Again, excesses appear more acute in the +1 frame. Unlike the previous model, +1 TAA is now the stop with the greatest number of genomes with excesses (298/694, 42.94%, *P *< 0.05, FDR correction) and greater than +1 TGA (256/694, 36.88%, *P *< 0.05, FDR correction). These +1 TAA excesses are highly restricted to the AT-rich genome and more generally have a significant negative correlation with GC content (ρ = −0.444, *P* < 2.2 × 10^−16^, Spearman’s rank correlation) (fig. 4, supplementary fig. 3, Supplementary Material online). In contrast, the number of genomes with significant excesses of +1 TAG (155/694, 22.33%, *P *< 0.05, FDR correction), +2 TAA (93/694, 13.40%, *P *< 0.05, FDR correction), and +2 TAG (42/694, 6.05%, *P *< 0.05, FDR correction) are remarkably low. Thus,  +1 seems to be the dominant signal, and signals for the most part are not associated with TAG.

It is again unclear whether the excesses reflect stop codon functionality. When compared with off-frame sense codons, both TAA and TAG have fewer genomes with significant excesses than either TAC or TAT. Excesses of TGC (+1: 599/694, 86.31%; +2: 557/694, 80.26%, *P *< 0.05, FDR correction) are the greatest of any TGN codon in either reading frame. Excesses of +1 TGG (271/694, 39.04%, *P *< 0.05, FDR correction) and +2 TGT (384/694, 55.33%, *P *< 0.05, FDR correction) are also greater than TGA in the respective frames.

### +1 TAA Demonstrates Evidence of OSC Selection at Synonymous Sites for Amino Acid Repeats Whose Codons Present the Opportunity to Encode an OSC

Results of the above simulation, which is arguably the most realistic determination of the null model, are suggestive but come with caveats, given the excess of OSCs. However, this null model also has limitations. First, we have to make presumptions about the realism of synonymous site selection. For example, if there are subtle location-specific codon usage biases or context-dependent mutational biases, these are likely to overcome any selection for OSCs. The model does not respect differential codon usage biases throughout the CDS nor motif or domain-specific codon usage biases, for example, the bias toward A to disrupt messenger RNA (mRNA) stability at 5’ ends (Gu et al. 2010 b; Kudla et al. 2009; Bentele et al. 2013). Furthermore, in assuming each synonymous site is under selection for OSCs, this model assumes selection pressures are of equal strength at all synonymous sites, which is unlikely to be the case.

Given the these issues, we propose a further test that might better control for amino acid order, codon usage biases, and highly regionalized effects, but one that has a more limited sample size. We can ask whether the synonymous codons used in localized sequence contexts encode OSCs when given the opportunity. We isolated any repeat of two isoleucine (codons ATA, ATC, ATT) or valine (codon GTA, GTC, GTG, GTT) amino acids, followed by amino acids whose codon starts with either C or T. In this way, we isolate sequences in which the first codon always has the opportunity to yield an OSC, followed by a second codon, encoding an identical amino acid that strictly cannot. Any regionalized biases are thus minimized while ensuring the amino acid requirement and hence direction of codon usage bias remains identical. If OSC selection constrains codon choice, we predict a stronger bias toward A-ending synonyms for the first codon of the repeat than the second. For example, A use in the sequence 5’-ATH | ATH | YNN-3’ should be greater at site 3 than 6 to encode +1 TAA. ATG has no synonyms, and therefore +1 TGA cannot be examined. We perform paired tests between usage within each genome to control for intragenome localized mutational biases but also to negate effects of intergenome compositional biases. We cannot control the mutational bias (or motif selection) owing to interactions between sites 3 and 4 and sites 6 and 7, but otherwise all other context features are preserved.

**Fig. 5.**
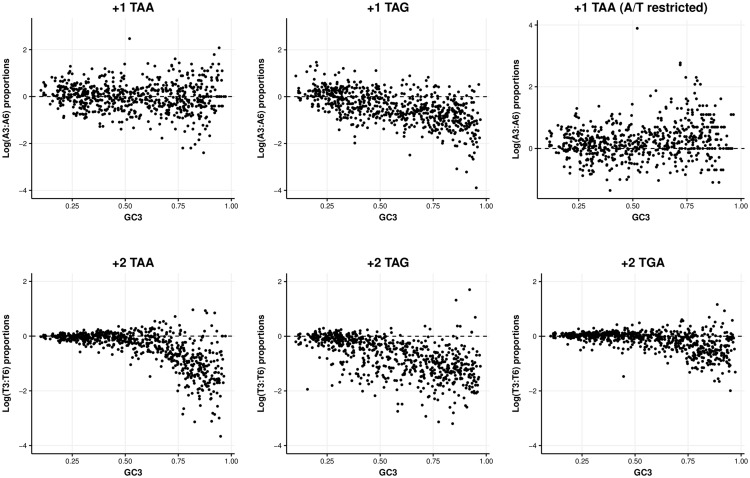
—Log ratios between the A use at synonymous sites of amino acids whose codons when repeated can generate an OSC. Correlations are significantly negative in each case (*P* < 0.05, Spearman’s rank correlations), suggesting A use at the third site decreases compared with the sixth, as GC mutational biases make encoding OSCs more difficult. When codons are restricted to only A/T ending synonyms, +1 TAA demonstrates a significant positive correlation with GC content (ρ = 0.160, *P* = 4.827 × 10^−5^, Spearman’s rank correlation).

Again, the signals are ambiguous. We find no significant difference between the use of A at sites 3 and 6 for +1 TAA encoding sequences (*P* = 0.215, paired Wilcoxon signed rank test). If synonymous sites are being selected for to preserve OSCs, we expect site 3 to be more resistant to mutational pressures than site 6. Thus, as GC3 content increases, we expect relatively little change in A3 but a reduction in A6 giving a positive correlation between A3: A6 and GC content. This is not the case—correlations are significantly negative for possible +1 TAA encoding sequences (ρ = −0.097, *P* = 0.012, Spearman’s rank correlation) (fig. 5).

This negative correlation might imply that the uncontrolled mutation bias difference (A3: A4 versus A6: A7, difference) is not to be overlooked. However, for this test, GC3 content is not consistent and allows comparisons between ATA and ATC. When GC3 content is controlled by only considering codons using A/T at their synonymous site, A3 use is significantly greater than A6 use (*P* < 2.2 × 10^−16^, paired Wilcoxon rank-sum test, mean proportion of sequences with A: site 3 = 0.278; site 6 = 0.208). Individually, 475/694 (68.44%) genomes have greater A3 use. Furthermore, the correlation between GC3 and A3/A6 correlations is now significantly positive (ρ = 0.160, *P* = 4.827 × 10^−5^, Spearman’s rank correlation). Thus, synonymous codon usage is consistent with +1 TAA selection after GC control.

We apply the same test to valine repeats that have the potential to encode +1 TAG. Unlike +1 TAA-encoding sequences, we find A3 use significantly reduced when all valine codons are considered (*P* < 2.2 × 10^−16^, paired Wilcoxon signed rank test, mean proportion of sequences with A: site 3 = 0.137; site 6 = 0.156) and when only GTA and GTT are considered (*P* = 6.129 × 10^−5^, paired Wilcoxon signed rank test, mean proportion of sequences with A: site 3 = 0.313; site 6 = 0.329). Correlations are significantly negative between GC3 content and A3: A6 usage in both cases (All codons: *ρ *= −0.585, *P* < 2.2 × 10^−16^, Spearman’s rank correlation; GTA/GTT: ρ* *= −0.143, *P* = 1.77 × 10^−4^, Spearman’s rank correlation).

Thus, it appears synonymous codon usage is consistent with OSC selection in the specific case of +1 TAA, although motif effects and subtle mutational biases are hard to eliminate as explanations. Employing similar tests for T use for all +2 OSC encoding sequences provides no evidence consistent with OSC selection, nor does a general hypothesis that considers all stop codons and frames together (supplementary result 3, Supplementary Material online).

### +1 TGA Densities Are Significantly Reduced in Genomes Where TGA Does Not Function as a Stop Codon, However Both +1 TAA and +1 TAG Densities Are Also Reduced

Although our models present excesses of OSCs in some instances, can we attribute them to stop codon function? The excess of off-frame sense codons suggests that simply looking for an excess of OSCs may be naive. An alternative approach is to consider the subset of prokaryotes (*Entomoplasmatales* and *Mycoplasmatales*) in which TGA is recoded to tryptophan, eliminating stop functionality (Bove 1993). If excesses are due to termination functionality, any off-frame TGA selection should be weaker in these genomes. Further, if terminating frameshift events is of such cellular importance, this recoding should result in compensatory increases of TAA and TAG due to the impaired termination ability. We refer to recoded genomes as “table 4” genomes and those using the standard genetic code as “table 11” genomes using National Centre for Biotechnology Information (NCBI) naming convention. Indeed, there would appear to be weaker +1 TGA selection (supplementary fig. 4, Supplementary Material online) with most table 4 genomes demonstrating negative excesses in our simulations. It is, however, important to compare actual OSC frequencies between genomes using alternative translation tables. Any differences attributable to GC mutational biases (i.e., AT-rich table 4 genomes are likely to have increased OSC densities by chance) are minimized by performing loess regressions and comparing residuals between the two genetic codes.

The OSC densities of stop codons combined are significantly reduced for table 4 genomes when +1 and +2 frames are considered together (*P* = 5.572 × 10^−4^, Kruskal–Wallis rank sum test of residuals; table 4 mean residual (MR) = −5.487, table 11 MR = 0.046). Results are similar when reading frames are considered separately (+1: *P* = 5.624 × 10^−4^, Kruskal-Wallis rank sum test of residuals, table 4 MR = −2.617, table 11 MR = 0.029; +2: *P* = 8.406 × 10^−4^, Kruskal–Wallis rank sum test of residuals, table 4 MR = −2.870, table 11 MR = 0.017). This is not entirely unexpected—even if TAA and TAG are somewhat increased there may not be full compensation for the loss of TGA.

Are these reduced OSC densities attributable to loss of TGA stop functionality? Contrary to expectation, off-frame TGA densities are significantly increased in the +2 frame (*P* = 0.002, Kruskal–Wallis rank sum test of residuals; table 4 MR = 1.113, table 11 MR = −0.011) supporting the excesses in simulation models (supplementary fig. 4, Supplementary Material online). Despite reduced mean residuals, +1 TGA densities are not significantly reduced (*P* = 0.125, Kruskal–Wallis rank sum test of residuals; table 4 MR = −0.233, table 11 MR = −0.001). However, given negative excesses from simulation models (supplementary fig. 4, Supplementary Material online) and these reduced residuals, the lack of table 4 genomes may be limiting. To provide a richer data set, we therefore incorporated all table 4 genomes from our initial data set prior to phylogenetic filtering, increasing the table 4 sample to 93 genomes. We accept that this introduces a degree of nonindependence and bias by including many *Mycoplasmas* (see supplementary table 1, Supplementary Material online, for breakdown of genomes).

**Fig. 6.**
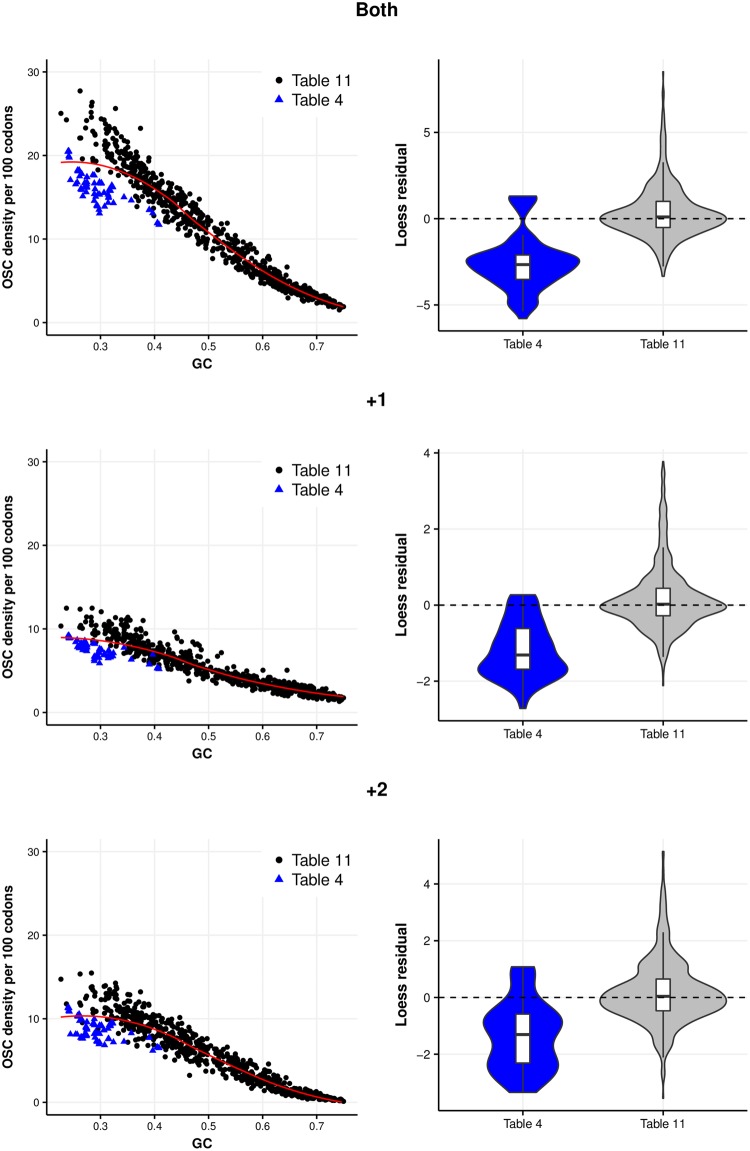
—OSC densities are reduced in table 4 genomes when compared with table 11 genomes in each alternative reading frame. Violin plots of the loess regression residuals highlight the reduced residuals for OSC densities in table 4 genomes.

With this increased data set, combined OSC densities in table 4 genomes remain significantly reduced when +1 and +2 frames are considered together (*P* < 2.2 × 10^−16^, Kruskal–Wallis rank sum test of residuals; table 4 MR = −2.509, table 11 MR = 0.363), in the +1 frame (*P* < 2.2 × 10^−16^, Kruskal–Wallis rank sum test of residuals, table 4 MR = −1.171, table 11 MR = 0.176) and the +2 frame (*P* < 2.2 × 10^−16^, Kruskal–Wallis rank sum test of residuals, table 4 MR =−1.337, table 11 MR = 0.187) (fig. 6). Specifically, although +2 TGA use remains significantly increased (*P* = 1.57 × 10^−9^, Kruskal–Wallis rank sum test of residuals; table 4 MR = 0.328, table 11 MR = −0.055), +1 TGA densities are significantly reduced (*P* = 2.091 × 10^−7^, Kruskal–Wallis rank sum test of residuals; table 4 MR = −0.174, table 11 MR = 0.023). Thus, consistent with previous results, any selection for OSCs is likely to be operating predominantly in the +1 frame and +1 TGA use appears to be reduced in table 4 genomes.

**Fig. 7.**
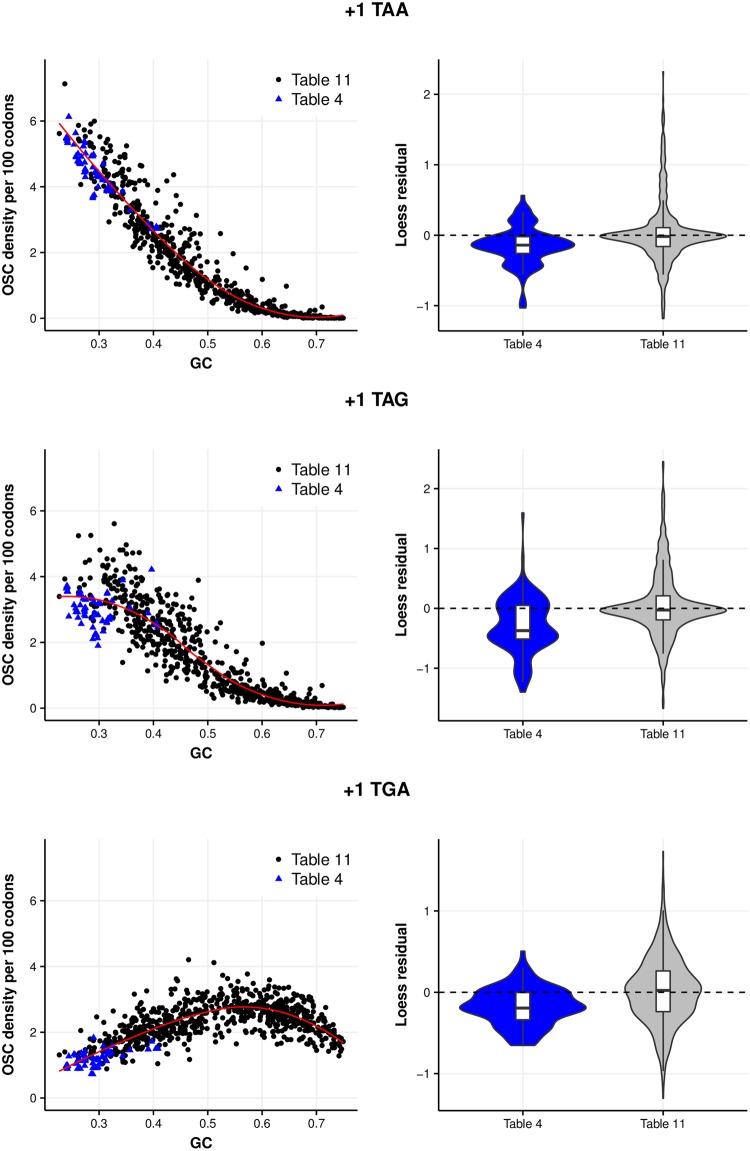
—OSC densities for table 4 genomes are reduced for each of the stop codons in the +1 frame. Violin plots of the loess regression residuals confirm the reduced densities of each OSC.

Without considering the context of this reduced excess, it is difficult to determine whether this is related to lost termination function. Are TAA and TAG densities increased to compensate? Results indicate this is not the case. Both +1 TAA (*P* = 3.107 × 10^−6^, Kruskal–Wallis rank sum test of residuals; table 4 MR = −0.149, table 11 MR = 0.028) and +1 TAG (*P* = 4.355 × 10^−8^, Kruskal-Wallis rank sum test of residuals; table 4 MR = −0.284, table 11 MR = 0.048) densities are significantly reduced in table 4 genomes (fig. 7). When restricting table 4 genomes to include only 9 *Mycoplasma* genomes (matching the total for the next most common genus *Spiroplasma* to reduced bias, see Methods) in which selection against TGA should be weakest, we obtain similar results (supplementary table 2, Supplementary Material online).

Given densities of other OSCs are not increased, we ask whether off-frame TAN and TGN densities are more generally reduced. Both +1 TAC (*P* = 1.14 × 10^−12^, Kruskal-Wallis rank sum test of residuals; table 4 MR = −0.170, table 11 MR = 0.020) and +1 TAT (*P* = 1.582 × 10^−13^, Kruskal–Wallis rank sum test of residuals; table 4 MR = −0.277, table 11 MR = 0.038) densities are also significantly reduced in table 4 genomes. Results are similar using the restricted *Mycoplasma* data set (supplementary table 2, Supplementary Material online). Thus, reduced TAA and TAG densities may not be termination-function related but rather a consequence of weakened selection for alternative constraints that affects all off-frame TAN codons. Alternatively, table 4 genomes may not exploit OSCs as a frameshift termination mechanism to the same degree, given termination capacity is reduced. These reduced densities dismiss the notion of increased compensatory selection.

For TGN codons, while there is no significant difference between +1 TGC densities (*P* = 0.101, Kruskal–Wallis rank sum test of residuals) or +1 TGT densities (*P* = 0.290, Kruskal–Wallis rank sum test of residuals), +1 TGG densities are significantly reduced (*P* = 0.003, Kruskal–Wallis rank sum test of residuals; table 4 MR = −0.137, table 11 MR = 0.015). For +1 TGC and +TGT, results using the restricted *Mycoplasma* data set are similar (supplementary table 2, Supplementary Material online) although +1 TGG densities are not significantly different (*P* = 0.257, Kruskal-Wallis rank sum test of residuals). Unlike TAN codons, it would be difficult to conclude that reduced TGA densities are attributable to reduced TGN densities but rather toward possible reduced TGR densities or reduced exploitation of OSCs in general. Differences between +1 TGG results when only *Mycoplasma* genomes with reduced negative TGA selection are included and when all are included could suggest that as +1 TGG densities are increasingly affected by the selection against +1 TGA (for codons encoding +1 TGA, G is the nucleotide most likely under selection, which also exists at the second position of +1 TGG). If +1 TGA has been selected against for sufficiently long, it is possible that +1 TGA and +1 TGG reach an equilibrium, whereby densities of both are reduced despite only TGA function being lost.

### A Refined Version of the Ambush Hypothesis

One might reasonably suggest that the above evidence only adds to the uncertainty of data related to the ambush hypothesis and highlights the sensitivity of the tests to small assumptions about how to test against a null. What is clear is that the ambush hypothesis cannot unambiguously explain OSC usage in all bacterial genomes. However, the data are such that we also cannot easily dismiss the hypothesis that no genome selects for OSCs. Importantly, there is a considerable overlap in the number of genomes with significant +1 excesses for both the codon shuffle model and synonymous site randomization model (+1 TAA: 90.60%, +1 TAG: 76.84%, +1: TGA: 67.84%, percentages of genomes in the model with most excesses that also have significant excesses in the model with fewer excesses), suggesting the signals we observe for both models are genuine. Prima facie these results appear to contradict the ambush hypothesis, as frameshift tracts should on average be shorter in AT-rich genomes (Warnecke et al. 2010; fig. 2). Thus, if there were to be a refined version of the hypothesis, it would need to explain why AT-rich genomes appear to be more associated with an excess. There is a possible (post hoc) refined version of the hypothesis that we suggest is worth considering and that makes some testable predictions.

### AT-Rich Genomes Have Higher Frameshift Rates, Consistent with the Refined Model

We (and others) (Tse et al. 2010 and Morgens et al. 2013) have assumed that the ambush hypothesis predicts greater excess from null in GC-rich genomes, as post-frameshift tract lengths in these genomes will be longer. However, this is only half of the equation. The other critical component is the rate at which frameshifts occur. If the rate of frameshifting is higher in AT-rich genomes, selection for OSCs could be higher, refining our model to predict absolutely higher rates, per base pair, in AT-rich genomes. We can test whether AT-rich genomes have higher rates of frameshifting in silico.

Previous evidence suggests that the composition of the tRNA repertoire is important in determining translational accuracy (Baranov et al. 2004; Shah and Gilchrist 2010; Warnecke, et al. 2010), with frameshift-susceptible codons decoded by rarer tRNAs (Curran and Yarus 1989; Sipley and Goldman 1993; Lainé, et al. 2008) and potentially struggling to meet stringent proofreading demands (Ieong et al. 2016). Enriching the tRNA repertoire correlates with reduced frameshift susceptibility (Warnecke et al. 2010). The susceptibility and cost of frameshifting, associated with tRNA abundance and diversity, may therefore be important in determining OSC frequency. The “process cost of accidental frameshift” model (Warnecke et al. 2010) incorporates tRNA information to calculate the susceptibility and cost of frameshifting.

We find the distribution of correlations between median CDS frameshift cost and OSC density approximately even around 0 (supplementary fig. 5*A*, Supplementary Material online). However, genomes where these correlations are positive are typically AT-rich (ρ = −0.353, *P* < 1.618 × 10^−8^, Spearman’s rank correlation). Thus, despite the on average reduced pre- and post-frameshift tract lengths (Warnecke et al. 2010; fig. 2), frameshifting cost appears to correlate with OSC density.

**Fig. 8:**
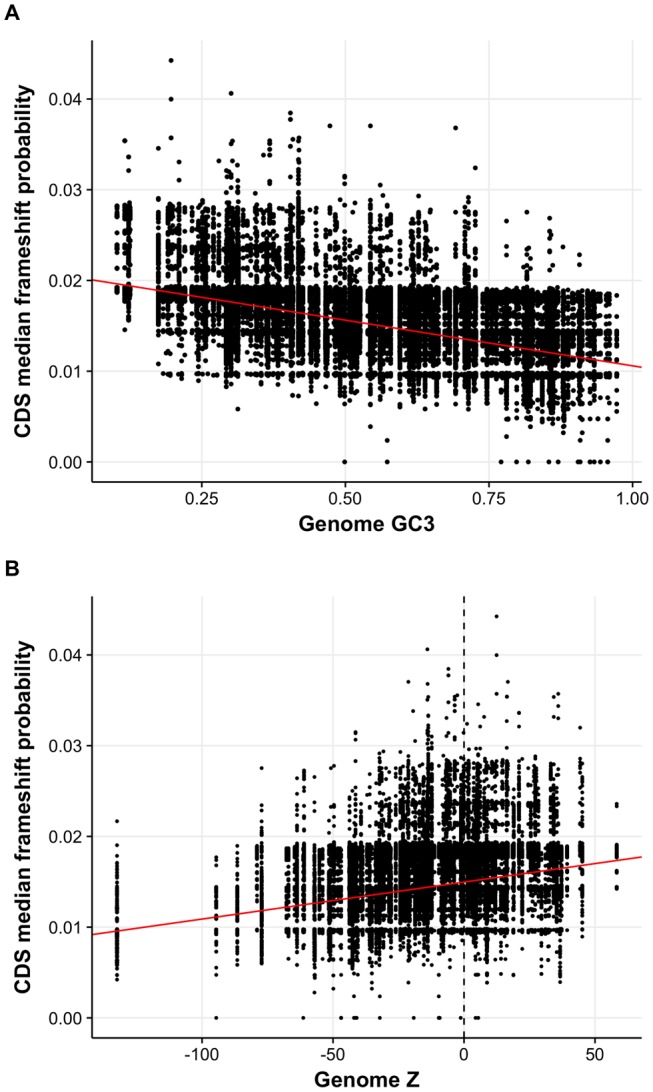
*.—(a)* The median probability of frameshifting decreases with increasing GC3 content. *(b)* Genomes with excesses of OSCs for the synonymous site model tend to have higher +1 frameshift probabilities, suggesting the frequency of OSCs and susceptibility of frameshifting are linked.

Are these increased OSC densities compensating for increased costs due to an increased propensity to frameshift? This appears to be the case, as AT-rich genomes seem more susceptible to frameshifting (ρ* *= −0.660, *P* < 2.2 × 10^−16^, Spearman’s rank correlation) (fig. 8*A*). Deviations from null (Z scores) are positively correlated with the susceptibility to frameshifting (codon shuffle: estimate: 0.150, *P* < 2.2 × 10^−16^; synonymous site simulation: estimate: 0.176, *P* < 2.2 × 10^−16^, Spearman’s partial correlations) (fig. 8*B*) and not a result of GC-content biases that may increase both frameshift susceptibility and OSC excess. This suggests that our explanation for the connection between AT-richness and OSC excess as a signal of selection in the refined model may have some virtue. In short, in genomes where frameshifting rates are high, tract lengths are typically short and OSCs in excess. Where tract lengths are long, an alternative general strategy to reduce frameshifting rates is the better strategy.

We note that a significant problem faced with this type of analysis is that we must make generalizations in order to compare between genomes. For instance, Warnecke et al. (2010) outline that codon–anticodon interactions are invariably generalizations, as tRNA decoding capacity cannot be predicted from sequence information alone. Furthermore, the effects of modifications to anticodon residues and tRNAs on decoding capacity (Cochella and Green 2005; Daviter, et al. 2006; Grosjean, et al. 2010) are likely to be genome specific. Thus, although results establish a relationship between signatures of OSC selection and frameshift probability, more in-depth conclusions regarding the extent to which OSCs are under selection should be considered in the knowledge of these limitations.

### A Refined Model Still Leaves Observations Unexplained

Given the above result, we suggest that the refined model may have some validity. However, although it is to a large degree a post hoc model, it fails to explain everything. Two results post the most obvious problems. First, why do we see so many biases of sense codons with similar nucleotide composition out of frame? Second, why is there a dearth of all off-frame stop codons in the table 4 genomes that do not employ TGA?

Regarding the second of these, had we observed an excess of +1 TAA but not TAG, this would have been consistent with the refined model, but we do not. However, the refined model makes no pretense to suppose all genomes cope with frameshifts by use of OSCs. By virtue of using a different code, table 4 genomes can be automatically considered to be somewhat exceptional. Indeed, selection pressures experienced by these organisms associated with their particular ecological niches (Bove 1993) may also be unusual. Another possibility is the weakened purifying selection attributable to smaller effective populations (*N*_e_) of table 4 genomes. However, if a universal GC to AT mutation bias exists (Lind and Andersson 2008; Hershberg and Petrov 2010), GC content should act as a reasonable proxy for low *N*_e_ (many AT-rich bacterial genomes likely have low *N*_e_). Thus, although reduced *N*_e_ may contribute, it is unlikely to explain the overall trends we observe.

Interestingly, we notice both TGA and TGG have similar numbers of genomes with off-frame excesses in our simulation models. Coupled with the results of table 4 genomes, this suggests excesses of TGA may not be related to termination function. In the refined model, increased densities of +2 TGA in the table 4 genomes support this notion, suggesting that some excesses are not associated with stop functionality but either reflect chance or missing layers of complexity not accounted for in our simulations. There may, for example, be constraints on protein-level motifs, or at the DNA or RNA level, coupled to localized selection for optimal codon usage that distorts out-of-frame usage as an incidental side consequence. For this reason, we remain skeptical that the ambush hypothesis, even in its refined form, commands any strong support at present. This being said, the fact some sense codons are enriched out of frame does not itself demonstrate that stop codon enrichment out of frame is not owing to stop functionality, but rather there might be an alternative unknown explanation. Thus, while both of these unexplained features are not obviously consistent with the refined model, neither are they lethal to it.

## Discussion

The notion that OSC selection should constrain sequence evolution to compensate for frameshift errors is logical. More recently, Morgens et al. (2013) demonstrated the initial result on which the ambush hypothesis was founded (Seligmann and Pollock 2004) is not robust to compositional control. Furthermore, this initial evidence only weakens after multiple correction testing (supplementary result 4, Supplementary Material online). However, an alternative approach using simulated sequences from Markov models identifies many genomes with an excess of OSCs (Tse et al. 2010; Morgens et al. 2013). An underlying issue with these models is their inability to strictly maintain amino acid frequencies, amino acid order, and codon usage frequencies. Under real evolutionary constraints, such flexibility is unlikely to be permitted and not realistic. Thus, the motivation of this paper was to establish the extent, if any, to which OSCs drive sequence evolution in a more realistic simulation framework and when microscale position effects are controlled.

We proposed and tested a series of simulation approaches, none of which control for all possible biases, but with each reaching similar conclusions (see supplementary table 2, Supplementary Material online, for summaries), the numbers of genomes with significant excesses are modest, often under 50%; genomes with an excess of OSCs tend to be AT-rich; and not all stop codons nor reading frames are equally affected. A post hoc model makes sense of these observations, but the predictions of this model regarding different handling of TGA and TAA compared with TAG and the preponderance of +1 frameshifts remain to be tested.

An important consequence of the refined model is that naively assuming GC-rich genomes bear greater frameshift costs does not account for more complex frameshift dynamics. Citing a positive correlation between GC content and any excess of OSCs as evidence consistent with OSC selection as in previous studies (Tse et al. 2010; Morgens et al. 2013), even if further analyses are not consistent with selection, is likely to be too simplistic. To more comprehensively quantify the cost of both frameshift errors and errors in general, it is important to consider complex relationships between error frequency and the selective constraints imposed to mitigate any costs.

The structure of the refined model more broadly considers frameshift control in a framework, whereby two distinct strategies have evolved and have different usage in different genomes. In one case, frameshifts are, on average, very damaging due to long frameshift tract lengths (GC-rich genomes). In this instance, a general reduced frameshifting rate is selectively advantageous which in turn reduces the selective pressure to incorporate any given OSC (although downstream of particularly frameshift-prone sites might be an exception). At the limit, if the frameshift rate could be reduced to zero, there would be no requirement for or selection for OSCs. Conversely, in other genomes (AT-rich), the average frameshift has little cost as tract lengths are naturally short. Here, selection cannot act to generally reduce frameshift rates, as there is likely be little return on investment of such a reduction for a given cost. However, even in these genomes, there will remain sites where by chance, tract lengths are long. In these sites, there could then be selection—given the high frameshifting rates—for OSCs. Thus, in this two-mode framework, we might expect more OSC excesses in AT-rich genomes and not as usually asserted in GC-rich genomes, although strategies are likely to be highly genome specific (as evidenced by negative excesses in many genomes).

One interesting notion arising from this framework is the coevolution of frameshift rates and OSCs. Whether proposed frameshift rate increases are due to weakened purifying selection in genomes with reduced *N*_e_ (assuming GC-rich genomes have larger effective population sizes), or whether the nucleotide content of AT-rich genomes naturally encoding greater numbers of OSCs means frameshifts are less costly, the ability to prevent frameshifting itself appears to be relaxed in AT-rich genomes. Parenthetically, error frequency may be the principal determinant of the strength of selection for OSCs in these genomes with this framework providing another possible example, whereby selection may be stronger in response to increased error rates when populations are small (Wu and Hurst 2015). In genomes where this frameshift error rate is reduced, or alternative pressures exert stronger selection on the CDS, the ability to maintain OSCs within CDSs may be significantly reduced and not a viable frameshift control strategy leading to significant depletions of OSCs. Indeed, other selective pressures, such as those imposed by environmental constraints (the ability to incorporate new DNA via off-frame recombination in metabolically versatile bacteria, or prevent recombination in more stable symbionts may be imperative to genetic adaptation; Wong et al. 2008), may also be important in determining the degree of OSC selection.

We also question why genomes tend to use TGA and TAA as OSCs. While TGA is the weakest of the stops (and prone to read-through) (Meng et al. 1995; Wei et al. 2016), TGA and TAA are unique in the specificity of release factors (RFs) decoding the stop codons: RF2 decodes both TAA and TGA (Kisselev 2002). RF2 in combination with RF3 is implicated in post peptidyl transfer quality control, ensuring more efficient termination at tRNA/mRNA mismatch complexes and proposed to participate in ribosome rescue (Zaher and Green 2009; Vivanco-Domínguez et al. 2012; Petropoulos et al. 2014). Specific capabilities of RF2 may therefore make TAA and TGA more suitable to frameshift termination, rather than the efficiency of termination of the stop codons themselves and predicts that captured frameshifts are more likely processed by the RF2/RF3 complex. In addition, minimal TAG excesses may possibly reflect avoidance of complementary GATC DNA motifs found frequently in nonrandom clusters on the bacterial chromosome (Touzain, et al. 2011).

One consistency is the bias toward excesses seen for +1 but not for the +2 frame. Here we can only conjecture that frameshifting, by accident, occurs predominantly in the +1 slippage mode. We can speculate that as translation occurs in the 5’ to 3’ direction, the molecular mechanics required to halt and reverse the direction of translation to the first nucleotide of a −1 frameshift, already held in the P-site, are likely to be more complex and require greater energy than for a ribosome to skip to the +1 frame in the same direction. Thus, accidental +1 frameshifts may be more frequent and require greater OSC control, although this is only speculation without comprehensive frameshift rate data and would no doubt benefit from molecular frameshift data. This should be experimentally testable. Our refined model is therefore one in which the genomes, stop codons, and reading frames are important factors in OSC selection.

### Problems Defining the Null

One of the lessons of the analysis presented here is that the meaning of a deviation from null is hard to interpret, not least because the results are dependent upon the definition of the null. Aside from the issue of which model is the most appropriate, we have looked for deviations at the genome level and not at the gene level. As OSC selection is likely to be sequence and context specific, it is also worth considering whether investigating OSC selection at the genome level is the most appropriate. For instance, Bertrand et al. (2015) have demonstrated no evidence consistent with OSC selection in the polyketide synthase (PKS) gene in fungi. Furthermore, sequences with differing levels of frameshifting are commonplace in coding regions of *E. coli* (Gurvich et al. 2003). As the information-carrying capacity of CDSs is limited, competing selection pressures providing more beneficial and selectable fitness advantages will be favored. Any selection for OSCs is likely to be one of several competing pressures, with OSC selection therefore potentially undetectable at whole genome scales.

Equally, a more appropriate approach may be to consider the single gene level, as selection may be stronger and more detectable in subsets of genes and avoided in others. For example, one might, at first sight, expect stronger selection in highly expressed genes. This hypothesis, however, has the caveat that highly expressed genes are likely to be composed of codons less susceptible to frameshifting (i.e., matching common tRNAs) and therefore not require OSC selection. The latter case, at least for +1 frameshifts for which this framework is most applicable, seems appropriate (supplementary fig. 6, Supplementary Material online). Alternatively, for genes overly susceptible to frameshifting, such as those incorporating mononucleotide repeats (Coenye and Vandamme 2005), OSCs provide an attractive strategy which tRNA selection is unable to regulate. Extending research to determine whether OSCs have important evolutionary implications at a single gene scale would help to inform us whether OSCs have useful applications in, for example, transgene design.

We also highlight two further limitations of our approach. First, an assumption of our models is that OSCs are indeed selected for. However, it is also known that organisms in all kingdoms utilize frameshifting to increase coding capacity to translate multiple proteins from the same CDS, for example the gag-pol protein (Jacks et al. 1988; Dulude et al. 2002) or in autoregulatory feedback systems (Baranov et al. 2002; Betney et al. 2010) via programmed frameshifting (Farabaugh 1996; Dinman 2012; Ketteler 2012). In such instances, the null expectation should not be selection for OSCs but rather strong avoidance selection. Even with the knowledge of well-annotated programmed frameshifts, it would be difficult to define how a null sequence with no selection should be composed. Our analyses cannot account for such programmed frameshifting without first removing CDSs where these frameshifts occur. The highly site-, context-, and CDS-specific nature of programmed frameshifts are, however, unlikely to greatly influence our conclusions.

Second, we assume that regardless of sequence context an OSC can function as a stop codon. Put differently, our null deviations are defined with respect to OSC number rather than OSC efficiency. There are, however, likely to be many alternative factors influencing the efficiency of terminations both for regular stop codons and for OSCs. For example, we assume that upon entering the ribosome A-site, an OSC functions as regular stop codon and has the same ability to recruit release factors. The nucleotide context surrounding stop codons, particularly the nucleotide following the stop codon, is also an important determinant of termination efficiency and read through (Poole et al. 1995; Tate et al. 1996; Mottagui-Tabar and Isaksson 1997; Namy et al. 2001; Cridge et al. 2006; Wei and Xia 2017). An initial analysis of the nucleotide 3’ of OSCs indicates no such bias (supplementary fig. 7, Supplementary Material online). In *E. coli*, the cooperation of chemical properties to the penultimate two amino acids in the nascent peptide to form secondary structures can also determine termination efficiencies (Mottagui-Tabar et al. 1994; Björnsson et al. 1996). Any analyses that can further establish the extent to which the sequence context surrounding stop codons has on termination efficiency and the implications for OSCs may provide useful.

In summary, we propose that for the ambush hypothesis to be considered as having any validity, care is required in defining null expectations and that a more appropriate framework is one that considers not all genomes, not all stops, and not all alternative frames as equally relevant. Our modified framework holds promise, given its ability to predict higher frameshifting rates in genomes with high OSC excess but comes with unexplained features and caveats.

## Supplementary Material

Supplementary data are available at *Genome Biology and Evolution* online.

## Supplementary Material

Supplementary materials
