# Supplementary material for: Refining the Ambush Hypothesis: Evidence That GC- and AT-Rich Bacteria Employ Different Frameshift Defence Strategies
Source: Genome Biol Evol. 2018 Apr 2;10(4):1153–73. doi: 10.1093/gbe/evy075 (PMC5909447; doi:10.1093/gbe/evy075)
Supplement: Supplementary materials [file evy075_supp.zip › Supplementary_figures_tables.docx]

# Supplementary Figures

Supplementary Figure 1: Mean OSC densities for *E*. coli in the codon shuffle model. Mean densities vary little beyond 100 repeats.

Supplementary Figure 2: Violin plots for OSC excesses in the each of the reading frames for the codon shuffle model. Genomes with significant positive excesses are typically AT-rich, particularly for TAG in all reading frames. Interestingly, the GC content of genomes with significant excesses of TGA are more similar to those without significant excess, suggesting that selection to incorporate off-frame TGA can overcome the restrictions of reduced AT-rich codons that make up OSCs in GC-rich genomes.

Supplementary Figure 3: Violin plots for OSC excesses in the each of the reading frames for the synonymous site model. GC content of genomes with significant positive excesses are extremely similar to those found for the codon shuffle model. Significant positive excesses are skewed towards the AT-rich genomes.

Supplementary Figure 4: Off-frame TGA densities of table 4 genomes for each simulation model. Table 4 genomes appear to have less +1 TGA than expected in the +1 frame.


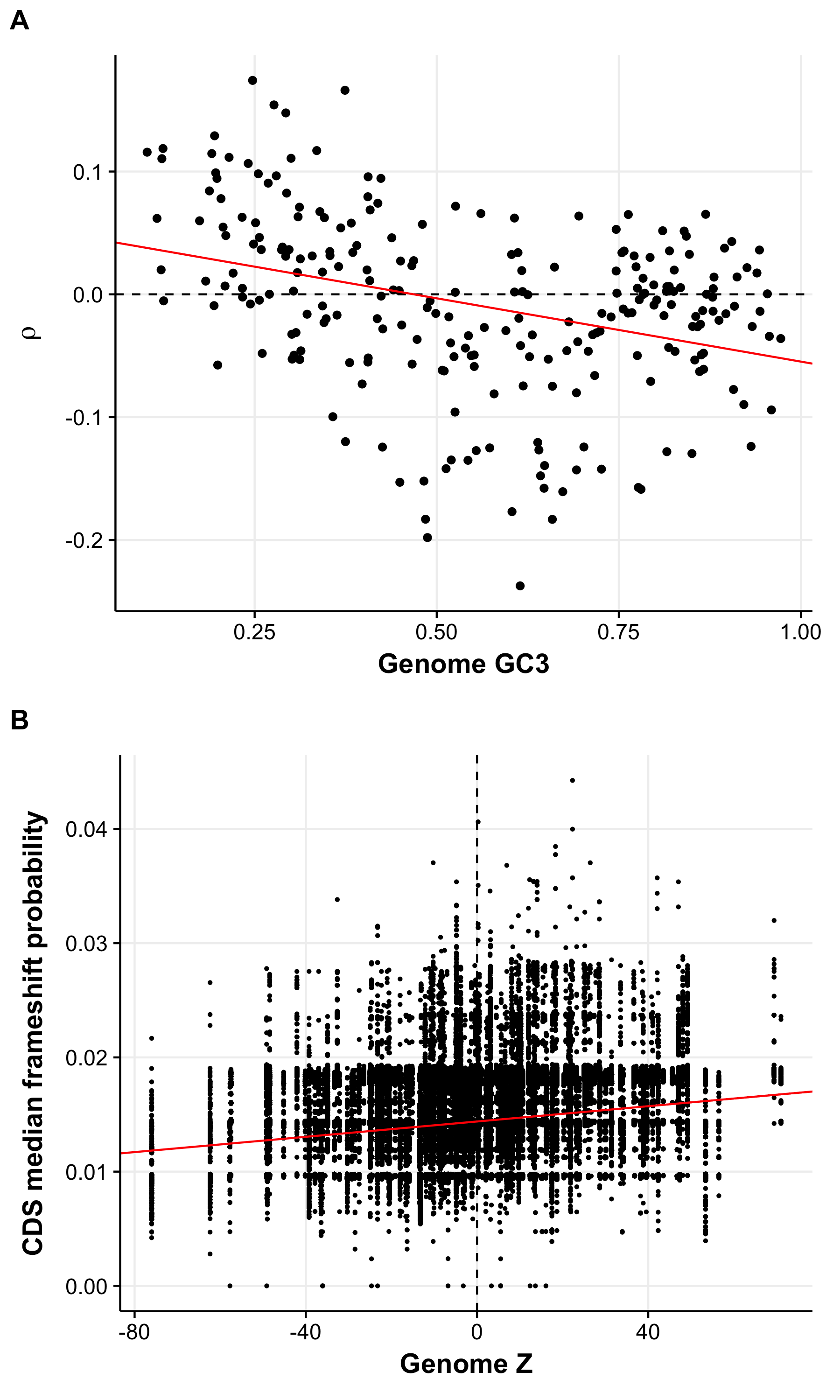


Supplementary Figure 5: A) Correlation between genome GC3 content and the genome correlation between the median cost of frameshifting and OSC density for each CDS. Genomes with a positive correlation between OSC density and frameshift costs are typically AT-rich, B) Genomes with excesses of OSCs for the codon shuffle model tend to have higher +1 frameshifts probabilities.

Supplementary Figure 6: OSC densities in the +1 frame for genes with higher CAI (as a proxy for gene expression) are lower than for genes with lower CAI. These differences are significantly different (*P* = 1.214 × 10^-9^, Kruskal-Wallis rank sum test of loess regression residuals). This supports the hypothesis that highly expressed genes are less susceptible to frameshifting and therefore requiring less OSCs.

Supplementary Figure 7: Log ratios comparing nucleotide use after a +1 OSC to nucleotide use in second codon position reveals no nucleotide bias.

# Supplementary Tables

Supplementary Table 1: A summary of the extended set of table 4 genomes

| Genus | Count | Percentage |
| --- | --- | --- |
| *Mycoplasma* | 76 | 81.72 |
| *Spiroplasma* | 9 | 9.68 |
| *Ureaplasma* | 4 | 4.30 |
| *Mesoplasma* | 2 | 2.15 |
| *Candidatus Mycoplasma* | 2 | 2.15 |

Supplementary Table 2: *P*-values for the Kruskal-Wallis rank sum test of residuals from a loess regression comparing codon densities in genomes using translation table 4 and table 11 when *Mycoplasma* genomes have been restricted. Mean residuals (MR) for the two translation tables are also shown.

| Codon(s) | Kruskal-Wallis *P*-value | Table 4 MR | Table 11 MR |
| --- | --- | --- | --- |
| Both frames, combined OSCs | < 2.2 × 10^-16^ | -4.083 | 0.197 |
| +1, combined OSCs | < 2.2 × 10^-16^ | -1.704 | 0.091 |
| +2, combined OSCs | 3.972 × 10^-16^ | -2.379 | 0.107 |
| +1 TAA | 2.848 × 10^-4^ | -0.271 | 0.017 |
| +1 TAC | 1.786 × 10^-6^ | -0.192 | 0.008 |
| +1 TAG | 5.839 × 10^-7^ | -0.440 | 0.033 |
| +1 TAT | 4.073 × 10^-9^ | -0.316 | 0.013 |
| +1 TGA | 0.032 | -0.133 | 0.003 |
| +1 TGC | 0.249 | -0.097 | 0.001 |
| +1 TGG | 0.257 | -0.125 | -0.002 |
| +1 TGT | 0.196 | -0.049 | -0.001 |
| +2 TGA | 7.77 × 10^-5^ | 0.493 | -0.030 |

Supplementary Table 2: Summary of expectations and results for each model.

| **Model** | **Expectations** | **Results** |
| --- | --- | --- |
| Codon randomisation within CDS | - Significant excess of OSCs when compared with the null (OSCs present due to chance dicodons) - Positive correlations between genome OSC excesses and GC content - Greater positive deviations from the null for OSCs when compared with sense codons - OSC excesses biased towards more efficient stop codons | - Number of genomes with significant excesses: max = 53.31% (+1 TGA), min = 6.34% (+2 TAG) - Significant excesses predominantly in the +1 frame - All OSCs with significant negative correlations with GC except +1 TGA (*ρ* = 0.036, *P* = 0.348) - Strong AT-bias for genomes with significant excesses, for each OSC - Greater excesses of TAC, TAT, TGC (+1, +2) and TGT (+2) - Excesses rank TGA > TAA > TAG (+1, +2) |
| Synonymous site randomisation within coding blocks | - Significant excesses of OSCs when compared with null (synonymous sites are not under selection to encode OSCs) - Positive correlations between genome OSC excesses and GC content - Greater positive deviations from the null for OSCs when compared with sense codons - OSC excesses biased towards more efficient stop codons | - Number of genomes with significant excesses: max = 52.59% (+2 TGA), min = 6.05% (+2 TAG) - Significant excesses predominately in the +1 frame - All OSCs with significant negative correlations with GC - Strong AT-bias for genomes with significant excesses, for each OSC - Greater excesses of TAC, TAT, TGC +1, +2), TGG (+1) and TGT (+2) - Excesses rank TAA > TGA > TAG (+1), TGA > TAA > TAG (+2) |
| Synonymous codon randomisation permitting interchange  between codon blocks | - Significant excesses of OSCs when compared with null (synonymous codon use is not determined by the ability to encode an OSC) - Positive correlations between genome OSC excesses and GC content - Greater positive deviations from the null for OSCs when compared with sense codons - OSC excesses biased towards more efficient stop codons | - Number of genomes with significant excesses: max = 52.02% (+2 TGA), min = 6.20% (+2 TAG) - Significant excesses predominantly in the +1 frame (Supplementary Result 2) - All OSCs with significant negative correlations with GC - Strong AT-bias for genomes with significant excesses, for each OSC - Greater excesses of TAC, TAT, TGC (+1, +2), TGG (+1), TGT (+2) - Excesses rank TAA > TGA > TAG (+1), TGA > TAA > TAG (+2) |
| OSC encoding amino acid repeats | - Significant increase in use of synonyms that encode OSCs for the first codon when compared with the second (which strictly cannot encode an OSC) - Positive correlations between GC content and the site 3:site 6 ratio of use of the OSC facilitating nucleotide | - Only in the case of +1 TAA (isoleucine repeat) and only when synonymous site restricted to A/T - Only +1 TAA with a significant positive correlation after restriction to only A/T at synonymous sites |
| Table 4 genome comparison | - Reduced off-frame TGA densities in table 4 genomes - Possible increased compensatory TAA and TAG off-frame densities in table 4 genomes | - Reduced +1 TGA, TAA and TAG densities in table 4 genomes - Reduced densities of all +1 TAN codons in table 4 genomes - Reduced densities of +1 TGR codons in table 4 genomes - Increased +2 TAA, TAG and TGA densities in table 4 genomes |
