# Supplementary material for: Refining the Ambush Hypothesis: Evidence That GC- and AT-Rich Bacteria Employ Different Frameshift Defence Strategies
Source: Genome Biol Evol. 2018 Apr 2;10(4):1153–73. doi: 10.1093/gbe/evy075 (PMC5909447; doi:10.1093/gbe/evy075)
Supplement: Supplementary materials [file evy075_supp.zip › Supplementary_results.docx]

# Supplementary Results

## Supplementary Result 1

**Markov modelling demonstrates significant positive correlations between OSC excesses and GC content consistent with previous studies**

We performed Markov modelling similar to that performed by Tse, et al. (2010) and Morgens, et al. (2013) in order to ascertain whether we could replicate previous excesses with our dataset. Tse, et al. (2010) report 99.1% of genomes with OSC excesses under the second-order model and 93.3% under the fifth-order model. Morgens, et al. (2013) report excesses in 83% of genomes analysed for both models. Our simulations report similar distributions of results, however using the Z metric we find no genomes with significant excess. We find OSC excesses in 677/694 (97.55%) genomes under second-order models and 689/694 (99.28%) under fifth-order models (Supplementary Result 1 Table 1).

When reading frames are considered individually, we find 661/694 (95.24%) and 683/694 (98.41%) genomes with significant excesses in the +1 frame using the second-order and fifth-order models respectively. In the +2 frame, 621/694 (89.48%) and 591/694 (85.16%) genomes exhibit significant excesses. Correlations between GC content and OSC excess are significant and positive for each model in each reading frame (second-order - both: *ρ* = 0.529, *P* < 2.2 × 10^-16^; +1: *ρ* = 0.450, *P* < 2.2 × 10^-16^; +2: *ρ* = 0.443, *P* < 2.2 × 10^-16^; fifth-order – both: *ρ* = 0.581, *P* < 2.2 × 10^-16^; +1: *ρ* = 0.279, *P* = 8.667 × 10^-14^; +2: *ρ* = 0.687, *P* < 2.2 × 10^-16^; Spearman’s rank correlations) (Supplementary Result 1 Figure 1) and are consistent with OSC selection as predicted by the ambush hypothesis and previously discussed (Morgens, et al. 2013; Tse, et al. 2010).

Supplementary Result 1 Figure 1: Correlations between GC content and OSC excesses (standard Z score) after CDS simulation using second-order three-periodic Markov models. Each reading frame, including when both are considered together, demonstrates significant positive correlations (*P* < 0.05, Supplementary Result 1 Table 1) with GC content, for both Markov models.

Do the sense codons demonstrate significant excesses greater than for OSCs, with significant positive correlations with GC content, as identified by Morgens, et al. (2013)?

Under the second-order model, +1 TAT (624/694, 89.91%) has a greater number of genomes with excess than +1 TAG (503/694, 72.48%) with a positive correlation between +1 TAA excesses and GC content (*ρ* = 0.596, *P* < 2.2 × 10^-16^, Spearman’s rank correlation), not displayed by either +1 TAA or +1 TAG. For the TGN codons, +1 TGA has the most genomes with excesses (513/694, 73.92%), although the correlation with GC content (*ρ* = 0.629, *P* < 2.2 × 10^-16^, Spearman’s rank correlation) is weaker than both +1 TGC (*ρ* = 0.736, *P* < 2.2 × 10^-16^, Spearman’s rank correlation) and +1 TGG (*ρ* = 0.728, *P* < 2.2 × 10^-16^, Spearman’s rank correlation). In the +2 frame, TAT (490/694, 70.61%) and TAC (298/694, 42.92%) have more excesses than TAG (284/694, 40.92%). The correlation between GC content and excesses is not significant for +2 TAA (*P* = 0.07, Spearman’s rank correlation), whilst both +2 TAC (*ρ* = 0.573, *P* < 2.2 × 10^-16^, Spearman’s rank correlation) and +2 TAT (*ρ* = 0.381, *P* < 2.2 × 10^-16^, Spearman’s rank correlation) have stronger significant positive correlations that +2 TAG (*ρ* = 0.313, *P* < 2.2 × 10^-16^, Spearman’s rank correlation). +2 TGA has the greatest number of excesses (666/694, 95.97%) compared with +2 TGC (570/694, 82.13%), +2 TGG (545/694, 78.53%) and +2 TGT (24/694, 3.46%).

Under the fifth-order model we find similar trends. +1 TAA (662/694, 95.38%) has greater number of genomes with excesses than other TAN codons, although the correlation with GC content is significantly negative (*ρ* = -0.355, *P* < 2.2 × 10^-16^, Spearman’s rank correlation) unlike the remaining TAN codons. +1 TAG has the fewest excesses of TAN codons (527/694, 75.94%). For +1 TGA codons, +1 TGA has the most excesses (603/694, 86.89%) and strongest positive correlation (*ρ* = 0.733, *P* < 2.2 × 10^-16^, Spearman’s rank correlation). For +2 TAN codons, TAA again has more excesses (682/694, 98.27%) with +2 TAT (664/694, 95.68%) having more than +2 TAG (657/694, 94.67%). +2 TGA also has greater excesses than any other +2 TGN codon (207/694, 38.90%).

Our results report distributions of excesses to Morgens, et al. (2013) with stop codons often with fewer genomes with excesses and less strongly positively correlated (negatively correlated) with GC content than for sense codons, advocating our choice of genomes. However, results should be interpreted with caution. For example, when both frames are considered together, TAC has significant positive correlations between GC content and OSC excesses and 97.12% and 96.54% of genomes with excesses for second-order and fifth-order models respectively. TAA, despite significant negative correlations (second-order model: *ρ* = -0.254, *P* = 1.305 × 10^-11^, Spearman’s rank correlation; fifth-order model: *ρ* = -0.355, *P* < 2.2 × 10^-16^, Spearman’s rank correlation) has excesses in 98.99% and 98.85% genomes. Results would therefore indicate stronger selection for TAA given the much-increased number of genomes with excesses. Thus, whilst it is important to consider other observation, the first consideration must be whether OSC frequencies deviate from the null frequencies.

Supplementary Result 1 Table 1: Summary of the Markov model simulation genome excesses for OSCs when considered together and individually for each reading. Sense codons are provided for comparison.

| Model |  | | 2^nd^ order Markov model | | 5^th^ order Markov model | |
| --- | --- | --- | --- | --- | --- | --- |
| Codon | **Reading frame** | | **# with excess** | **% with excess** | **# with excess** | **% with excess** |
| All stops | | **Both** | 677 | 97.55 | 689 | 99.28 |
| All stops | | **+1** | 661 | 95.24 | 683 | 98.41 |
| All stops | | **+2** | 621 | 97.55 | 591 | 85.16 |
| TAA | | **Both** | 687 | 98.99 | 686 | 98.85 |
| TAC | | **Both** | 674 | 97.12 | 670 | 96.54 |
| TAG | | **Both** | 665 | 95.82 | 606 | 97.32 |
| TAT | | **Both** | 684 | 98.56 | 644 | 92.80 |
| TGA | | **Both** | 309 | 44.52 | 400 | 57.64 |
| TGC | | **Both** | 327 | 47.12 | 203 | 29.25 |
| TGG | | **Both** | 333 | 47.98 | 57 | 8.21 |
| TGT | | **Both** | 321 | 46.25 | 10 | 1.44 |
| TAA | | **+1** | 678 | 97.69 | 662 | 95.39 |
| TAC | | **+1** | 652 | 93.95 | 627 | 90.35 |
| TAG | | **+1** | 503 | 72.48 | 527 | 75.94 |
| TAT | | **+1** | 624 | 89.91 | 609 | 87.75 |
| TGA | | **+1** | 513 | 73.92 | 603 | 86.89 |
| TGC | | **+1** | 444 | 63.98 | 252 | 36.31 |
| TGG | | **+1** | 452 | 65.12 | 69 | 9.94 |
| TGT | | **+1** | 11 | 1.59 | 23 | 3.31 |
| TAA | | **+2** | 527 | 75.94 | 682 | 98.27 |
| TAC | | **+2** | 298 | 42.92 | 647 | 93.23 |
| TAG | | **+2** | 284 | 40.92 | 657 | 94.67 |
| TAT | | **+2** | 490 | 70.61 | 664 | 95.68 |
| TGA | | **+2** | 666 | 95.97 | 270 | 38.90 |
| TGC | | **+2** | 570 | 82.13 | 181 | 26.08 |
| TGG | | **+2** | 545 | 78.53 | 113 | 16.28 |
| TGT | | **+2** | 24 | 3.46 | 243 | 35.01 |

## Supplementary Result 2

**Genomes exhibit minimal OSC excesses when given the flexibility in codon choice between multiple coding blocks permits a choice between synonymous codons that can and cant encode an OSC**

We consider a third simulation model, similar to our model which randomises synonymous sites. If we further permit changes between coding blocks, we can ask whether selection favours codons that encode OSCs if given the choice between codons that do and do not. For example, suppose the peptide sequence necessitates a valine followed by serine. If OSCs exert a strong enough selection pressure, we would expect preferential use of GTA or GTG valine codons followed by AGC or AGT serine codons as opposed to GTC or GTT and the T-starting serine codons to encode a +1 OSC. To consider selection to this effect, we randomised the use of synonymous codons throughout the genome, accounting for genome specific codon usage frequencies, controlling amino acid sequences and GC content whilst disrupting site-specific synonymous codon choice. OSCs generated from one-fold degenerate codons are not considered as randomisation has no effect on the identity of these codons.

Similar to the other models, evidence is not consistent with OSC selection. Only 84/694 (12.10%) genomes have significant excesses of OSCs (*P* < 0.05, FDR correction). This result is however, strongly influenced by the reduced excess in the +2 frame; only 107/694 (15.42%) genomes demonstrate significant excesses (*P* < 0.05, FDR correction) compared with 262/694 (37.75%) in the +1 frame (*P* < 0.05, FDR correction). Correlations between GC content and excesses are significantly negative for each reading frame (Supplementary Result 2 Table 1). The evidence to suggest CDSs favour codons that generate an OSC is weak and limited predominantly to the +1 frame, with significant excesses highly restricted to the AT-rich genomes (Supplementary Result 2 Figure 1).

Supplementary Result 2 Table 1: The number of genomes with significant out-of-frame excesses for different codons in the various reading frames when synonymous codons have been randomised. Spearman’s rank correlations between GC content and OSC excess, defined by the standard Z score are also shown.

| Codon | | Reading frame | | # with excess | % with excess | *ρ* | *P* |
| --- | --- | --- | --- | --- | --- | --- | --- |
| All stops | **Both** | | 84 | | 12.10 | -0.444 | < 2.2 × 10^-16^ |
| All stops | **+1** | | 262 | | 37.75 | -0.458 | < 2.2 × 10^-16^ |
| All stops | **+2** | | 107 | | 15.42 | -0.234 | 4.781 × 10^-10^ |
| TAA | **Both** | | 116 | | 16.71 | -0.513 | < 2.2 × 10^-16^ |
| TAC | **Both** | | 160 | | 23.05 | -0.051 | 0.176 |
| TAG | **Both** | | 90 | | 12.97 | -0.273 | 3.407 × 10^-13^ |
| TAT | **Both** | | 194 | | 27.95 | -0.364 | < 2.2 × 10^-16^ |
| TGA | **Both** | | 281 | | 40.49 | -0.336 | < 2.2 × 10^-16^ |
| TGC | **Both** | | 629 | | 90.63 | 0.595 | < 2.2 × 10^-16^ |
| TGG | **Both** | | 264 | | 38.04 | -0.416 | < 2.2 × 10^-16^ |
| TGT | **Both** | | 252 | | 36.31 | -0.345 | < 2.2 × 10^-16^ |
| TAA | **+1** | | 296 | | 42.65 | -0.437 | < 2.2 × 10^-16^ |
| TAC | **+1** | | 366 | | 52.74 | 0.581 | < 2.2 × 10^-16^ |
| TAG | **+1** | | 157 | | 22.62 | -0.322 | < 2.2 × 10^-16^ |
| TAT | **+1** | | 432 | | 62.25 | 0.404 | < 2.2 × 10^-16^ |
| TGA | **+1** | | 252 | | 36.31 | -0.169 | 7.942 × 10^-6^ |
| TGC | **+1** | | 596 | | 85.88 | 0.623 | < 2.2 × 10^-16^ |
| TGG | **+1** | | 269 | | 38.76 | -0.383 | < 2.2 × 10^-16^ |
| TGT | **+1** | | 105 | | 15.13 | -0.131 | 5.497 × 10^-4^ |
| TAA | **+2** | | 95 | | 13.69 | -0.308 | 1.496 × 10^-16^ |
| TAC | **+2** | | 146 | | 21.04 | -0.379 | < 2.2 × 10^-16^ |
| TAG | **+2** | | 43 | | 6.20 | -0.151 | 6.600 × 10^-5^ |
| TAT | **+2** | | 183 | | 26.37 | -0.491 | < 2.2 × 10^-16^ |
| TGA | **+2** | | 361 | | 52.02 | -0.249 | 3.367 × 10^-11^ |
| TGC | **+2** | | 557 | | 80.26 | 0.185 | 9.933 × 10^-7^ |
| TGG | **+2** | | 265 | | 38.18 | -0.209 | 3.156 × 10^-8^ |
| TGT | **+2** | | 381 | | 54.90 | -0.391 | < 2.2 × 10^-16^ |

Individually TGA demonstrates the greatest excesses when considered in both reading frames (Supplementary Result 2 Table 1). Few genomes have an excess of TAG in any frame. Each OSC, in each reading frame, demonstrates significant negative correlations with GC content (Supplementary Result 2 Figure 2, Supplementary Result 2 Figure 3). The number of genomes with significant excesses in the +1 frame is greatest for TAA (296/694, 42.65%, *P* < 0.05, FDR correction). In comparison, TAA use in the +2 frame is extremely reduced (95/694, 13.69%, *P* < 0.05, FDR correction), with +2 TGA having the highest number of genomes with excesses (361/694, 52.02%, *P* < 0.05, FDR correction).

For off-frame sense codons, TGC has an extremely high number of genomes with significant positive excesses in each frame (both: 629/694, 90.63%; +1: 596/694, 85.88%; +2: 557: 80.26%, *P* < 0.05, FDR correction) and is greater than TGA in each reading frame. Both TAC and TAT have greater excesses than TAA or TAG in any reading frame and show significant positive correlations with GC content in the +1 frame. Neither TAA nor TAG demonstrates significant positive correlations in any reading frame.

Given the flexibility of the model to allow for synonymous codon interchange within coding blocks for arginine and serine, we would have expected greater excesses of TAA and TGA in the +1 frame, or TAG in the +2 frame, given the ability of real coding sequences to encode an OSC simply by using the A-starting synonyms. This is not the case. For +2 TAG in particular, where the second codon in the encoding dicodon can only be either an AGR arginine or AGY serine codon, we find extremely low number of genomes with significant excesses (43/694, 6.20%).

As with other models, we have to recognise several limitations to this model. Selection pressures on the CDS resulting in local synonymous codon biases, for example to reduce 5’ mRNA stability (Bentele, et al. 2013; Goodman, et al. 2013; Gu, et al. 2010; Kudla, et al. 2009; Qing, et al. 2003) are likely to be stronger than for including an OSC. Selection for synonymous codons that encode OSCs is likely to be limited to sequence sites without additional requirements. This model also assumes sequences permit flexibility between synonyms from two coding blocks, which is unlikely to occur given a codon change of this type requires mutations at two positions of the codon. However, evidence from this model is not consistent with predictions for OSC selection.

Supplementary Result 2 Figure 1: Correlations between OSC excesses (Z) and GC content, when all genome stop codons are considered together, are significantly negative for each reading frame for a model in which synonymous codons are randomly simulated. Violin plots emphasise that genomes with significant excesses are typically AT-rich.

Supplementary Result 2 Figure 2: Correlations between genomes excesses (Z) and GC content are significantly negative for all stop codons in each reading frame when coding sequences are simulated by randomising synonymous codons and permitting changes between codon blocks.

Supplementary Result 2 Figure 3: Violin plots for OSC excesses in the each of the reading frames for the synonymous codon model. GC content of genomes with significant positive excesses are similar to those found in the codon shuffle model. Unlike the codon shuffle model, the GC content of genomes with significant positive excesses of TGA are more biased towards the AT-rich genomes, with only a subset of GC-rich genomes have excesses in the +1 frame.

## Supplementary Result 3

We can ask similar questions concerning localised synonymous site selection for OSCs using the sequences of amino acids repeats that provide the opportunity to encode +2 OSC, using asparagine for TAA (AAT, AAC), serine for TAG (AGT, AGC) and aspartic acid TGA (GAT, GAC). In these cases, site 3 T usages should be increased, although we are unable to control for GC3 content. However, T is used significantly less at site 3 in all cases (+2 TAA: *P* < 2.2 × 10^-16^; +2 TAG: *P* < 2.2 × 10^-16^; +2 TGA: *P* < 5.911 × 10^-9^, paired Wilcoxon rank sum tests). Moreover, correlations between GC3 content and log T3:T6 ratios are significantly negative in each case (+2 TAA: *ρ* = -0.642, *P* < 2.2 × 10^-16^; +2 TAG: *ρ* = -0.636, *P* < 2.2 × 10^-16^; +2 TGA: *ρ* = -0.513, *P* < 2.2 × 10^-16^, Spearman’s rank correlations).

We can test an overall hypothesis that synonymous codon usage is biased towards codons that generate OSCs if the following codon will allow by considering one-tailed tests all +1 and +2 contexts. This hypothesis is not supported (*P* ≈ 1, Fisher’s method combining one-tailed paired Wilcoxon rank sum tests). Thus, after minimising the potential effects that localised contexts may have had on our models, our evidence provides little support for any consistent genome wide OSC selection pressure, restricted to +1 TAA contexts.

## Supplementary Result 4

**Significant positive correlations between codon contribution to hidden stops are limited to AT-rich genomes following multiple correction testing and predicted by genome GC content**

Although it is has been shown that the method Seligmann and Pollock (2004) use for detecting OSC excesses is not appropriate (Morgens, et al. 2013), we replicated the analysis with our genome sample. Positive correlations were identified in 341/694 (49.13%) genomes of which 201 (28.96%) are significant, although we also find 141 (20.32%) significant negative correlations. However, Seligmann and Pollock (2004) make no mention of correction for multiple comparisons. When we perform such correction, we find only 121 (17.44%, FDR correction) genomes maintain significant positive correlations (Supplementary Result 4 Figure 1). Thus, the original evidence underpinning the ambush hypothesis is limited and weakened further after such control. Results therefore suggest that not only is the evidence for selection for OSCs determining codon usage weak (positive correlation in less than half of genomes) and inappropriate, but the strength of results are further weakened by further statistical analyses.

Supplementary Result 4 Figure 1: Significant positive Spearman’s rank correlations between genome codon usage and codon contribution to OSCs are highly restricted to AT-rich genomes after correction for multiple comparisons.

## References

Bentele K, Saffert P, Rauscher R, Ignatova Z, Bluthgen N 2013. Efficient translation initiation dictates codon usage at gene start. Mol. Syst. Biol. 9: 675.

Goodman DB, Church GM, Kosuri S 2013. Causes and Effects of N-Terminal Codon Bias in Bacterial Genes. Science 342(6157): 475-479.

Gu W, Zhou T, Wilke CO 2010. A Universal Trend of Reduced mRNA Stability near the Translation-Initiation Site in Prokaryotes and Eukaryotes. PLoS Comput Biol 6(2): e1000664.

Kudla G, Murray AW, Tollervey D, Plotkin JB 2009. Coding-Sequence Determinants of Gene Expression in Escherichia coli. Science 324(5924): 255-258.

Morgens DW, Chang CH, Cavalcanti ARO 2013. Ambushing the ambush hypothesis: predicting and evaluating off-frame codon frequencies in Prokaryotic Genomes. BMC Genomics 14: 418-418.

Qing G, Xia B, Inouye M 2003. Enhancement of translation initiation by A/T-rich sequences downstream of the initiation codon in Escherichia coli. J. Mol. Microbiol. Biotechnol. 6(3-4): 133-144.

Seligmann H, Pollock DD 2004. The ambush hypothesis: hidden stop codons prevent off-frame gene reading. DNA Cell Biol. 23(10): 701-705.

Tse H, Cai JJ, Tsoi H-W, Lam EP, Yuen K-Y 2010. Natural selection retains overrepresented out-of-frame stop codons against frameshift peptides in prokaryotes. BMC Genomics 11(1): 1-13.
